# Immunological profiling in type 2 diabetes mellitus and type 2 diabetic kidney disease: insights from single-cell LacNAc sequencing

**DOI:** 10.3389/fendo.2025.1550925

**Published:** 2025-08-11

**Authors:** Mengyun Xiao, Zigan Xu, Xiaohui Zhu, Jing Chen, Ru Wang, Yaxuan Wang, Xiang Gao, Shaodong Luan, Xiaoyan Pu

**Affiliations:** ^1^ School of Medicine, Qinghai University, Xining, Qinghai, China; ^2^ Department of Nephrology, Shenzhen Longhua District Central Hospital, Shenzhen, Guangdong, China; ^3^ Institute of Nephrology and Blood Purification, The First Affiliated Hospital of Jinan University, Guangzhou, Guangdong, China

**Keywords:** type 2 diabetes mellitus, diabetic kidney disease, neutrophil extracellular traps, net formation, ROS, oxidative stress, single-cell LacNAc sequencing

## Abstract

**Background:**

Diabetic kidney disease (DKD), a major complication of type 2 diabetes mellitus (T2DM), is the leading cause of end-stage renal disease (ESRD). Recently, the innate immune system, particularly neutrophils and the process of NET formation, has garnered significant attention for its role in the progression of T2DKD in patients with T2DM. However, the underlying mechanism remains unclear.

**Methods:**

We employed single-cell LacNAc sequencing (scLacNAc-seq) to characterize immune cell populations, glycosylation patterns, and functional alterations in peripheral blood mononuclear cells (PBMCs), focusing on low-density granulocytes (LDGs), from patients with T2DM and T2DKD versus healthy controls (HC). *In vitro* cultures of primary human neutrophils under high glucose and high glucose plus serum from patients with T2DKD were used to assess NET formation via myeloperoxidase (MPO) detection. Plasma levels of reactive oxygen species (ROS), CXCL8, CXCR2, MPO, and neutrophil elastase (NE) were quantified by ELISA.

**Results:**

Patients with T2DM and T2DKD showed increased LDG counts and glycosylation abundance in FOLR3- and PI3-expressing subclusters. Functional enrichment analysis of overlapping differentially expressed genes (DEGs) and subclusters revealed enrichment in NET formation pathways. *In vitro* studies promoted NET release, as evidenced by reduced intracellular MPO and elevated supernatant MPO under hyperglycemic conditions. Plasma ROS, CXCL8, CXCR2, MPO, and NE levels were elevated in patients with T2DM and T2DKD than in HCs. Furthermore, enhanced interactions between neutrophils and mononuclear phagocytes (MPs), primarily mediated by the CXCL8/CXCR2 axis, were observed.

**Conclusion:**

This study identifies immunological alterations in T2DM and T2DKD, implicating neutrophil-mediated inflammation and NET formation in T2DKD progression. Correlative data suggest that targeting ROS and the CXCL8/CXCR2 pathway may represent potential therapeutic directions, though preclinical validation is warranted.

## 1 Introduction

Diabetic kidney disease (DKD), a predominant complication arising from Type 2 diabetes mellitus (T2DM), stands as a significant contributor to end-stage renal disease (ESRD) (1). This condition exerts a profound negative impact on patients’ quality of life and represents a considerable economic burden on global healthcare systems (2).

The pathogenesis of DKD is multifactorial and involves hemodynamic changes, metabolic disorders, and chronic inflammation (3). Notably, the innate immune system plays notable role in T2DM and the progression to T2DKD (4). Neutrophils, the most abundant immune cells, have been implicated in T2DM complications, including T2DKD (5, 6), however, their functional heterogeneity and role in sterile inflammation remain underexplored.

As the first line of defense against pathogens, neutrophils release neutrophil extracellular traps (NETs), which are chromatin structures decorated with antimicrobial proteins such as myeloperoxidase (MPO) and neutrophil elastase (NE) (7). It is important to note that the term “NETosis,” which was previously used to describe NET formation, is no longer recommended. Current research indicates that NET formation can occur through two distinct pathways. One is a lytic process, while the other is a vital mechanism where neutrophils remain viable and functional after releasing NETs, as demonstrated by Dr. Paul Kubes’ group (8).

The induction of NET formation involves a complex network of molecular regulators. Reactive oxygen species (ROS) play a pivotal role in triggering the process (9). Upon activation, NADPH oxidase in neutrophils generates ROS, which then initiates a cascade of events leading to NET release (9–11). Peptidylarginine deiminase 4 (PAD4) is another key molecule (12). PAD4 catalyzes the citrullination of histone proteins in the nucleus, causing chromatin decondensation and facilitating the extrusion of NETs (11, 12). Gasdermin D, involved in pyroptosis, also contributes to the lytic pathway of NET formation by forming pores in the cell membrane (13, 14).

Dysfunctional NET formation contributes to tissue injury in diabetes and its complications by propagating inflammation and thrombosis (6, 15, 16). Critically, cytokines (e.g., TNF-α, IL-8) and metabolic stressors can “prime” neutrophils for enhanced NET generation (17). When neutrophils are pre-exposed to these cytokines, they become more responsive and release larger amounts of NETs upon subsequent stimulation (17, 18). This “priming” effect is an important aspect of the dysregulated immune response in inflammatory diseases.

Additionally, cellular senescence, which is associated with ageing, promotes dysregulated NET release (19). In chronic diseases, senescent cells secrete various factors that can activate neutrophils and disrupt the normal control mechanisms of NET formation, leading to excessive and potentially harmful NET release (19).

Sustained hyperglycemia in T2DM and T2DKD disrupts mitochondrial function, impairs the electron transport chain, and increases NADPH oxidase activity, leading to reactive oxygen species (ROS) overproduction (11, 20), a key inducer of NET formation (20).

Additionally, glycosylation, which is essential for protein structure and function, is altered under chronic hyperglycemia in T2DM and DKD (21). Changes in glycosylation patterns are associated with disease progression; for instance, a decrease in β-Gal, 2,6-sialic acid, and α-mannose, along with an increase in α1,6-fucose and GlcNAc levels in T2DM patients, correlates with heightened platelet reactivity and thrombosis risk (22). However, the impact of these glycosylation changes on neutrophil function and NET formation under hyperglycemic conditions has not been fully elucidated (23).

Crucially, in pathological states like T2DKD, a distinct low-density granulocyte (LDG) subset accumulates in the peripheral blood mononuclear cell (PBMC) fraction (24). LDGs exhibit enhanced NET-forming capacity and are implicated in autoimmune and metabolic diseases (25). In this study, we specifically analyzed LDGs isolated from the PBMC fraction to investigate their glycosylation profiles and NET-forming potential in T2DKD pathogenesis.

## 2 Aims

This study aimed to characterize disease-specific alterations in LDGs in the PBMC fraction of T2DKD patients—and their mechanistic contributions to disease progression. Using scLacNAc-seq, an innovative approach resolving glycosylation patterns and transcriptomes at single-cell resolution (26, 27), we profiled terminal GlcNAc exposure on immune cells to identify glycosylation signatures driving neutrophil dysfunction. Further, we investigated how hyperglycemia-induced ROS dysregulates NET formation in LDGs and evaluated the functional interplay between glycosylation changes, ROS, and NET-driven inflammation. Key findings were validated via enzyme-linked immunosorbent assay (ELISA) and functional assays.

## 3 Methods

### 3.1 Study design

Participants were recruited from three cohorts: HC, T2DM, and T2DKD. Whole blood samples were collected from each participant in EDTA-coated tubes. PBMCs were isolated from a subset of 10 samples per group for single-cell transcriptomic LacNAc sequencing and associated downstream analyses. Plasma was extracted from all blood samples to quantify the concentrations of ROS, C-X-C motif chemokine ligand 8 (CXCL8), C-X-C motif chemokine receptor 2 (CXCR2), MPO, and NE by ELISA.

Inclusion Criteria for T2DM Patients: a. confirmed diagnosis of T2DM based on prior medical records; b. at least two hemoglobin A1c (HbA1c) screenings >6.5% within the past year, with HbA1c >6.5% at the time of enrollment; c. urinary albumin-to-creatinine ratio (UACR) <30 mg/g creatinine; d. estimated glomerular filtration rate (eGFR) >90 mL/min. Exclusion Criteria: a. patients with primary hypertension; individuals with recent infections, history of immune-related diseases, history of infectious diseases, concurrent liver diseases, or other primary/secondary renal disorders.

Inclusion criteria for patients with T2DKD: a. confirmed diagnosis of T2DM based on prior medical records; b. HbA1c screenings >6.5% within the past year, with HbA1c >6.5% at the time of enrollment; c. UACR >30 mg/g creatinine; d. eGFR >30 mL/min. Exclusion criteria: patients with primary hypertension; individuals with recent infections, history of immune-related diseases, history of infectious diseases, concurrent liver diseases, or other primary/secondary renal disorders.

### 3.2 PBMCs isolation and single-cell suspension preparation

Isolated PBMCs were prepared for scLacNAc-seq as previously described (27, 28). Briefly, PBMC suspensions were cooled and filtered, and GEXSCOPE^®^ Red Blood Cell Lysis Buffer (RCLB, Singleron Biotechnologies Co., Ltd., Jiangsu, China) was added to remove the red blood cells. The mixture was incubated for 5–8 min and centrifuged at 350 g at room temperature for 3 min. The supernatant was discarded and the cells were gently resuspended in PBS (HyClone, GE Healthcare Co., Ltd., Pittsburgh, USA). A cell concentration of 1.5 × 10 –3.5 × 10^5^ cells/mL was achieved for loading onto the microchip. Cell viability was assessed microscopically after staining with trypan blue. If the cell viability was below 85%, a cell sorter was employed for healthy cell enrichment or dead cell depletion.

### 3.3 Glycosylation labeling

Single-cell suspensions from the previous step were subjected to LacNAc labeling using a specific DNA barcode with the ProMoSCOPETM Single Cell Glycosylation Detection Kit (Singleron Biotechnologies, Co., Ltd., Jiangsu, China) according to the manufacturer’s instructions. Glycosylation sites on the cell surface were labeled with tags containing poly A tails, unique oligonucleotide sequences, and chemical moieties that efficiently bind to glycosylation sites through fucosyltransferase catalysis. After three washes to remove excess labels, the cells were resuspended in PBS and adjusted to a final concentration of 1 × 10^6^ cells/mL. Subsequent operations were performed according to the manufacturer’s instructions (Singleron Biotechnologies, Co., Ltd., Jiangsu, China).

### 3.4 Transcriptome and glycosylation library construction

The prepared cell suspensions were loaded onto the microwell chip using the Singleron Matrix^®^ Single Cell Processing System. Following single-cell isolation, lysis, and barcoding, the barcoding beads were collected for reverse transcription and PCR amplification. After fragmentation, adapter ligation, and additional PCR, the amplified complementary DNA (cDNA) was constructed into a library compatible with the Illumina sequencing platform. The scLacNAc products were amplified via PCR to generate a sequencing library suitable for Illumina Novaseq 6000 with 150-bp paired-end reads. The individual libraries were diluted to 4 nM, pooled, and sequenced.

### 3.5 Single-cell RNA-seq and scLacNAc-seq library analysis

Barcodes and unique molecular identifiers (UMIs) for single-cell RNA-seq (scRNA-seq) were extracted and corrected from R1 reads. Adapter sequences and poly-A tails were trimmed from R2 reads using Cutadapt v3.7. The preprocessed R2 reads were aligned to the GRCh38 transcriptome using STAR v2.6.1b. Uniquely mapped reads were assigned to exons using FeatureCounts (v2.0.1). Successfully assigned reads with the same cell barcode, UMI, and gene were grouped to generate a gene expression matrix for further analysis. Concurrently, scLacNAc UMIs, identified with a 15-bp tag labeled at the glycosylation site on the cell surface, were extracted from R2 reads based on their position and compared with known glycosylation tag sequences. Tag sequences with a Hamming distance of less than 2 were considered valid. The number of UMIs for each cell barcode was counted to generate a single-cell glycosylated UMI expression matrix.

### 3.6 Quality control, dimension reduction, and clustering

Cells were filtered to include those with gene counts between 200 and 5,000 and UMI counts < 30,000. Cells with mitochondrial content greater than 20% were excluded. After filtering, 29,254 cells were used for the downstream analysis. Using gene expression data from each sample, Scanpy was used to identify cell clusters. Principal component analysis (PCA) was performed for normalization and dimension reduction, selecting the first 50 principal components for clustering using the Louvain algorithm. Uniform Manifold Approximation and Projection (UMAP) was employed for dimensionality reduction and visualization in two-dimensional space. Six cell clusters were identified based on the top ten primary marker genes and a resolution parameter of 1.2. LacNAc UMIs were detected and N-glycosylation abundance was normalized and evaluated using a centered log-ratio transformation for each sample.

### 3.7 Pathway enrichment analysis, differentially expressed genes identification, venn diagram, and gene-sets scoring

Kyoto Encyclopedia of Genes and Genomes (KEGG) pathway enrichment analysis was performed on sub-cell clusters FOLR3, PI3, CAMK1, CAM, ISG15, and MMP9, as well as for DEGs, using the ClusterProfiler (v3.10.1) R package. Pathways with a Benjamini–Hochberg adjusted P value of <0.05 were considered significantly enriched. Overlapping genes between T2DM *vs*. HCs and T2DKD *vs*. HCs were identified, and a Venn diagram was created using the Xiantao Xueshu online tool. Genes related to T2DM and T2DKD were obtained from GeneCards (https://www.genecards.org/) and filtered using a relevance score greater than five for gene set analysis. Subcell clusters of neutrophils were evaluated using UCell based on these gene sets, and UCell score distributions were visualized using UMAP. The stemness of neutrophil sub-cell clusters was assessed by calculating single-cell entropy, as previously described (27, 29). Gene sets related to aging were downloaded from GSEA (https://www.gsea-msigdb.org/gsea/index.jsp) and evaluated using UCell within neutrophil subcell clusters.

### 3.8 Pearson correlation

Pearson correlations were analyzed and scatter plots were generated using GraphPad Prism 8 (GraphPad Software, San Diego, USA, www.graphpad.com).

### 3.9 ScRNA-seq trajectory analysis

To identify different cell states and elucidate the sequential differentiation trajectory of neutrophil sub-cell clusters in HCs, T2DM patients, and T2DKD patients, Monocle3 (v0.1.3) was used to plot the sub-cell clusters onto a pseudotime trajectory. Genes with an adjusted *P* value of <0.05 were considered significantly changed and retained for further analysis. Additionally, Monocle3 was used to plot LacNAc UMIs onto the trajectory timeline.

### 3.10 Cell–cell interaction analysis

Cell–cell communication networks among sub-cell clusters of neutrophils and MPs were inferred through the CellPhoneDB (v2.1.2) repository, as described previously (27). NicheNet was used to assess the target genes of the ligand–receptor pairs among different cell clusters.

### 3.11 ELISA measurements from plasma samples

The plasma and cell supernatant concentrations of NE, and MPO-DNA were measured using ELISA kits (Guangzhou Aosila Biotechnology Co., Ltd., Guangzhou, China). Plasma concentrations of CXCL8 and CXCR2 were quantified using ELISA kits from Jingmei Biology Engineering Co., Ltd. (Jiangsu, China).

Human plasma ROS levels were quantified using a commercial ELISA kit (Guangzhou Bohui Biotechnology Co., Ltd., Guangzhou, China), according to the manufacturer’s protocol. Briefly: Standards (0–100 IU/mL) and plasma samples (diluted 1:5 in kit diluent; 10µL sample + 40µL diluent) were added in duplicate to the antibody-coated microplate. Blank wells contained diluent only. Horseradish peroxidase (HRP)-conjugate reagent (100 µL) was added to all wells. The plate was sealed and incubated (60 min, 37°C). Wells were aspirated and washed 5 times with Wash Buffer (400 µL per wash). Residual liquid was removed by tapping the inverted plate on absorbent paper. Chromogen solutions A and B (50 µL each) were added to each well. The plate was incubated protected from light (15 min, 37°C). Stop Solution (50 µL) was added to each well. Optical density (O.D.) was measured at 450 nm within 15 minutes using a microplate reader. A standard curve (O.D. 450 nm *vs*. standard concentration) was generated using 4-parameter logistic (4PL) regression. Sample ROS concentrations (IU/mL) were interpolated from the curve.

### 3.12 Human neutrophil isolation from peripheral blood

Peripheral blood was collected in EDTA/heparin-coated tubes and diluted 1:1 with PBS. Granulocytes were enriched via dextran sedimentation (6% dextran, 30–45 min incubation, RT) followed by density gradient centrifugation (Ficoll-Paque PLUS, 400 × g, 30 min, brake off). The granulocyte/RBC pellet was subjected to RBC lysis (ACK buffer, 10 min, RT), washed twice in PBS, and resuspended in RPMI-1640 supplemented with 10% FBS. Neutrophil purity (>95%) was confirmed by Giemsa staining, with viability >95% via trypan blue exclusion. All steps were completed within 3 hours to minimize activation.

### 3.13 Cell culture

Neutrophils were isolated from the blood samples of 10 healthy individuals and divided into three groups: control, high glucose (HG), and high glucose plus T2DKD serum (HG + T2DKD serum). T2DKD serum samples were collected from five patients with T2DKD, which were independent of the prior cohort. The serum was subsequently utilized to stimulate HK2 cells *in vitro*, thereby establishing a model mimicking the T2DKD-specific pathological microenvironment. The HG group received medium supplemented with 30 mmol/L glucose solution. The HG + T2DKD serum group received the same glucose supplementation along with 10% filtered serum from T2DKD patients, replacing fetal bovine serum. Photographs were taken under a microscope (Leica DMi1, Leica Microsystems, Wetzlar, Germany) at 0 and 30 h under different culture conditions. Cells and supernatants from each condition were collected after 30 h.

### 3.14 Western blot analysis

For WB analysis of MPO in neutrophils under different culture conditions, SDS-PAGE on a 10% gel was performed with 10 μg cell protein per lane (or maximal volume when protein <10μg). For the loading control, total protein was measured using the Revert Total Protein Stain (Licor, Lincoln, USA). Primary antibodies against human MPO and Actin were raised in rabbits at 59 and 42 kDa, respectively (Servicebio, Wuhan, China). HRP-labeled goat anti-rabbit antibody was used as the secondary antibody.

### 3.15 Statistical analysis

Data are presented as means ± SEM. Data were tested for normality with the Kolmogorov-Smirnov-Test, D’Agostino and Pearson omnibus normality test and Shapiro Wilk-Test. Variances were tested using the Bartlettś test for equal variances. Comparisons among data of HCs, patients with T2DM and patients with T2DKD were tested for significance using one-way ANOVA, and data from two groups were tested with unpaired Student’s t-test, Mann-Whitney U-test using GraphPad Prism 8, GraphPad Software (San Diego, USA, www.graphpad.com). Densitometric analysis of the WB was performed using ImageJ Version 1.50d (Wayne Rasband, USA, https://imagej.net/ij/download.html). Statistical significance was set at *P <*0.05.

## 4 Results

### 4.1 Clinical characteristics of the study cohort

Whole blood samples were collected from three distinct groups: HCs, patients with T2DM, and those with T2DKD. The exclusion criteria included recent infectious episodes, cancer, or a history of trauma to ensure the homogeneity of the study population. The final analysis included 20 participants per group. Plasma was separated from each sample for ELISA validation. The study design is shown in **Supplementary Figure 1**.

As detailed in **Table 1**, the mean age of the T2DM and T2DKD cohorts was higher than that of HCs. Additionally, the T2DKD group had a significantly longer duration of diabetes than the T2DM group. Hypertension, particularly systolic hypertension, was more pronounced in patients with T2DKD than in both HCs and T2DM patients (**Table 1**). A comprehensive overview of the clinical and laboratory parameters of all the participants is provided in **Table 1**. Although neutrophil counts did not differ significantly across the groups, an increasing trend was observed in the T2DKD group. Furthermore, the neutrophil-to-lymphocyte ratio (NLR) was higher in T2DKD patients than in HCs, suggesting a potential inflammatory bias in this cohort.

**Table 1:** Clinical characteristics and laboratory parameters of HC, T2DM, and T2DKD groups.

Parameters		HC (n=20)	T2DM (n=20)	T2DKD (n=20)
Gender	Male (%)	50	50	70
	Female (%)	50	50	30
Age (years)		31 ± 7	68 ± 4 #	61 ± 7 #
History of diabetes (years)		0	7 ± 5	16 ± 10 #*
Systolic blood pressure (mmHg)		111 ± 8	131 ± 25	160 ± 17#*
Diastolic blood pressure (mmHg)		71 ± 7	72 ± 9	83 ± 11#
Glycated hemoglobin (%)		4.4 ± 0.3	7.3 ± 1.1#	8.0 ± 1.6#
White blood cell count (10E9/L)		5.8 ± 0.4	6.4 ± 0.4	6.4 ± 0.4
Neutrophil count (10E9/L)		4.0 ± 0.5	4.0 ± 0.3	4.5 ± 0.3
Neutrophil/lymphocyte ratio		2.9 ± 0.4	2.6 ± 0.3	4.6 ± 0.5*
Plasma C-reactive protein level (mg/L)		4.2 ± 0.4	4.1 ± 0.5	3.5 ± 0.7
Plasma albumin (g)		45 ± 4	40 ± 4	35 ± 6#
eGFR (mL/min)		126 ± 15	121 ± 16	52 ± 15 #*
Urine albumin/creatinine (mg/g crea)		11 ± 3	18 ± 6	245 ± 232#*

#Indicates a statistically significant difference compared to the HC group. *Indicates a statistically significant difference compared to the T2DM group (*P* < 0.05).

### 4.2 Elevated neutrophil counts and enhanced glycosylation in patients with T2DM and T2DKD: insights from scLacNAc-seq analysis

**Figure 1:**
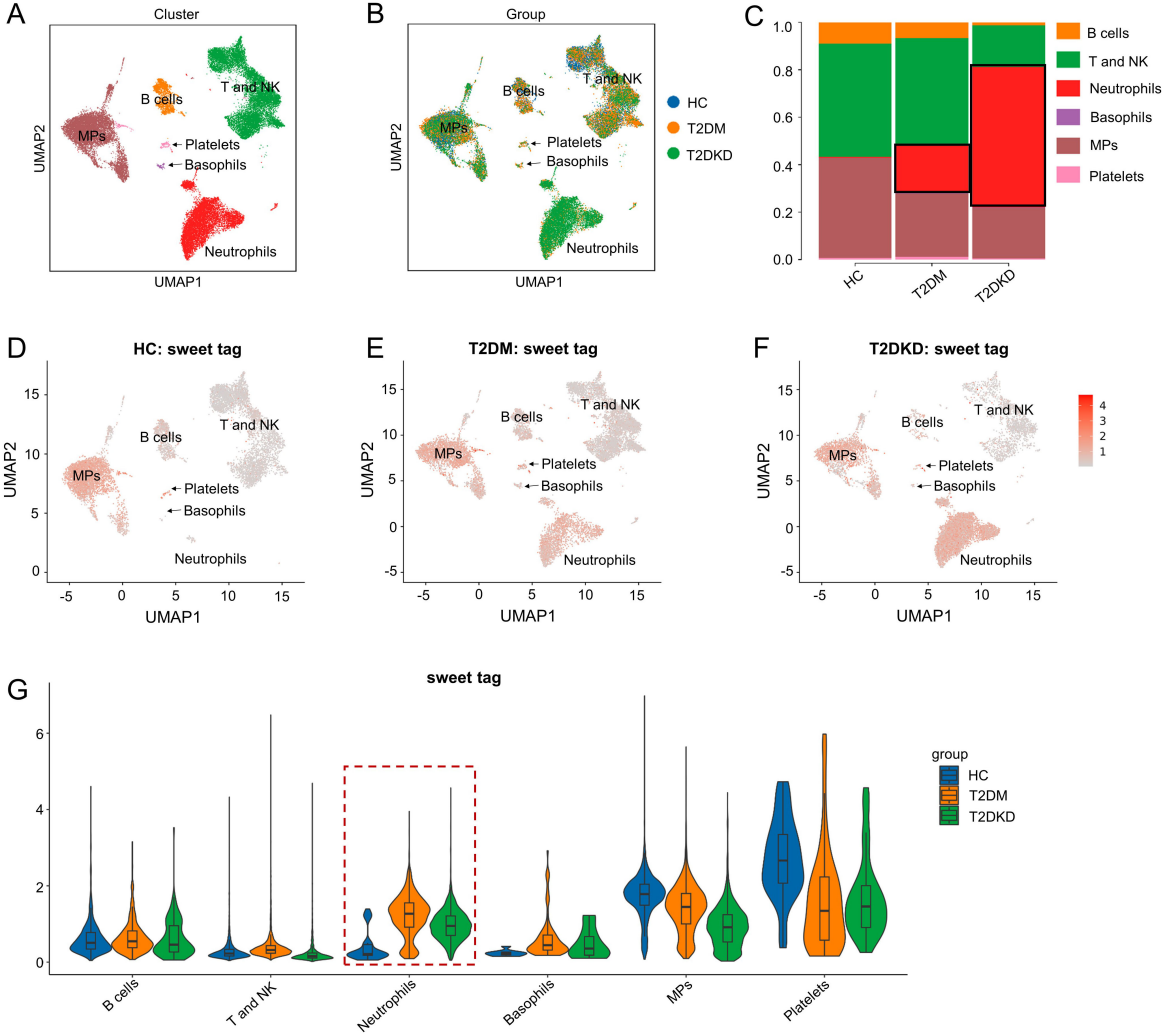
Cell clusters and glycosylation abundance identified by scLacNAc-seq. **(A)** A total of 29,254 cells were identified through scLacNAc-seq, categorized into six cell types: **(B)** cells, T and NK cells, Neutrophils, Basophils, MPs, and Platelets. **(B)** UMAP dimension reduction illustrates the cell distribution across the three groups. **(C)** Bar plots depict the ratio of each cell cluster within each group. **(D–G)** UMAP dimension reduction **(D–F)** and violin plots **(G)** demonstrate glycosylation abundance for each cell cluster in the HC, T2DM, and T2DKD groups, respectively.

Data obtained from scLacNAc-seq were processed and analyzed according to the established quality control criteria (**Supplementary Table 1**). An automated annotation method, supplemented by manual review and calibration, was used to assign identity labels to each cell based on single-cell expression profiles and the expression of specific cell marker genes (**Supplementary Table 2**, **Supplementary Figure 2A**). In total, 29,254 cells were identified and categorized into six distinct cell clusters: B cells, T and NK cells, neutrophils, basophils, mononuclear phagocytes (MPs), and platelets (**Supplementary Table 2**, **Figures 1A–C**). Detailed information regarding these cell clusters, including marker genes, cell counts, and percentages, is summarized in **Supplementary Table 2**.

Both the number and proportion of neutrophils were elevated in patients with T2DM and T2DKD compared to those in HCs, while the number and proportion of MPs were significantly reduced (**Supplementary Table 2**, **Figures 1A–C**). Specifically, neutrophil counts and proportions were 32 cells (0.38%) in HCs, 2,198 cells (20.09%) in patients with T2DM, and 5,968 cells (60.58%) in patients with T2DKD (**Supplementary Table 2**, **Figures 1A–C**). In contrast, the number and proportion of MPs were 3,458 (40.87%) in HCs, 2,792 (25.52%) in patients with T2DM, and 1,958 (19.88%) in patients with T2DKD. Additionally, glycosylation levels in neutrophils were found to be significantly increased in patients with T2DM and T2DKD compared to those in HC (**Figures 1D–G**).

Analysis of DEGs revealed an upregulation in the expression of CXCL8 and CXCR2, which are pivotal chemokines and receptors instrumental in neutrophil recruitment. This upregulation was observed when comparing patients with T2DM to HCs (**Supplementary Figure 2B**) as well as between patients with T2DM and T2DKD (**Supplementary Figure 2C**). The consistent elevation of these markers in patients with T2DM and T2DKD is in concordance with the noted increase in neutrophil count.

A total of 702 overlapping DEGs were identified between HCs and patients with T2DM as well as between patients with T2DM and T2DKD, as illustrated in the Venn diagram (**Supplementary Figure 2D**). Functional enrichment analysis of these DEGs revealed an enrichment in the NET formation pathway (**Supplementary Figure 2E**).

### 4.3 Sub-cell clusters analysis of neutrophils reveals enhanced NET formation in patients with T2DM and T2DKD

**Figure 2:**
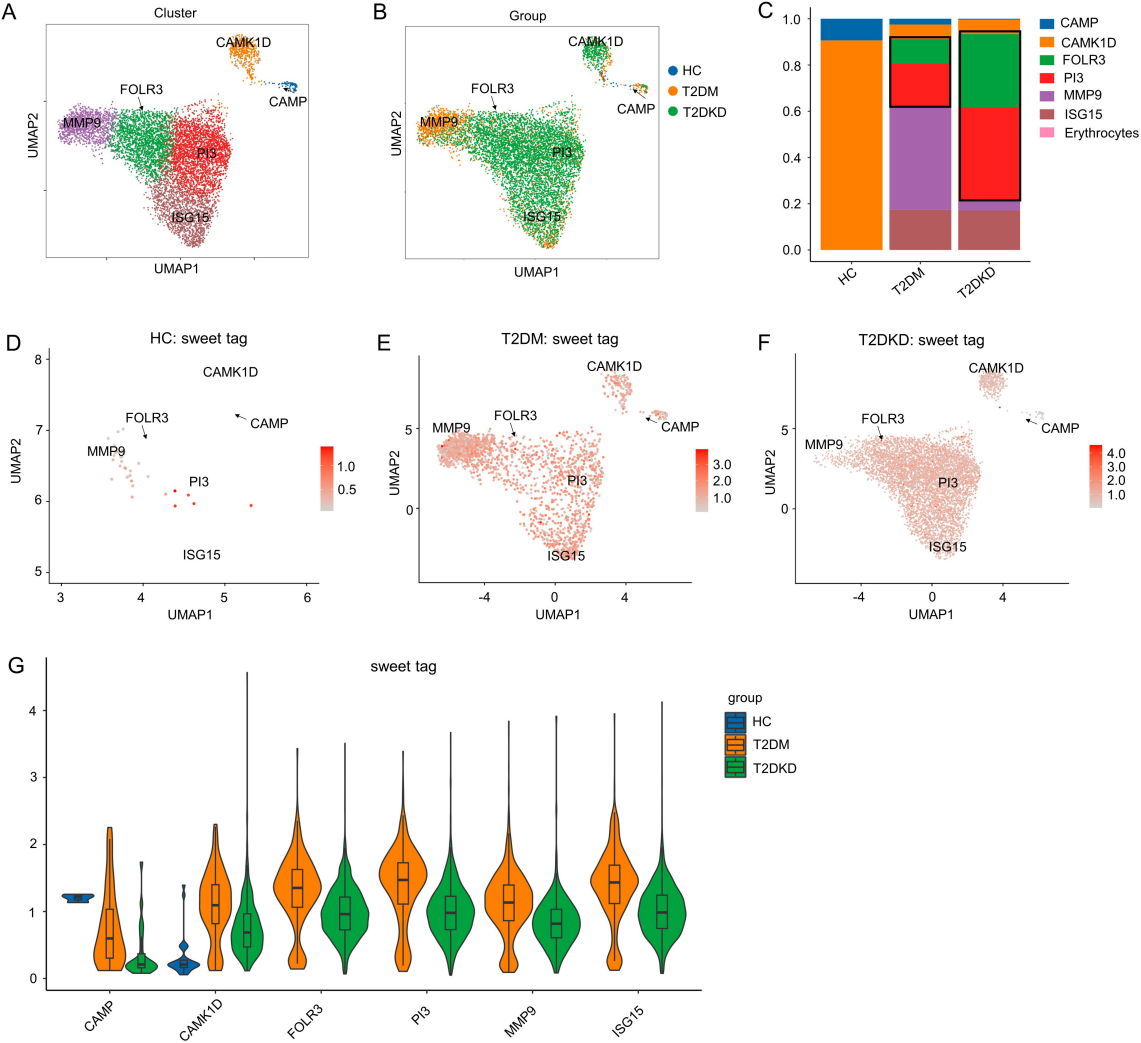
Sub-cell clusters and glycosylation abundance of neutrophils. **(A, B)** A total of 8,198 neutrophil cells were identified, categorized into six sub-cell clusters across the groups. **(C)** Bar plots display the ratio of each cell cluster across the groups. **(D–G)** UMAP dimension reduction **(D–F)** and violin plots **(G)** illustrate glycosylation abundance for each cell cluster in the HC, T2DM, and T2DKD groups, respectively.

To delve deeper into the roles of elevated neutrophils and reduced MPs in T2DM and T2DKD, we conducted a sub-cell cluster analysis, categorizing neutrophils and MPs into six and four distinct sub-populations, respectively. Sub-clustering of neutrophils identified six distinct subpopulations, based on the most prominently expressed genes, including CAMP, CAMK1D, PI3, MMP9, ISG15, FOLR3 in T2DM and T2DKD patients (**Figures 2A–C**, **Supplementary Table 3**). Notably, FOLR3 and PI3 subclusters were undetectable in HCs, while CAMP predominated in this group (**Figure 2C, G**). This absence suggests that FOLR3/PI3 subpopulations may emerge specifically during diabetic progression. Moreover, the abundance of glycosylation was elevated in the FOLR3 and PI3 sub-cell clusters in patients with T2DM and T2DKD relative to that in HCs (**Figures 2D–G**).

**Figure 3:**
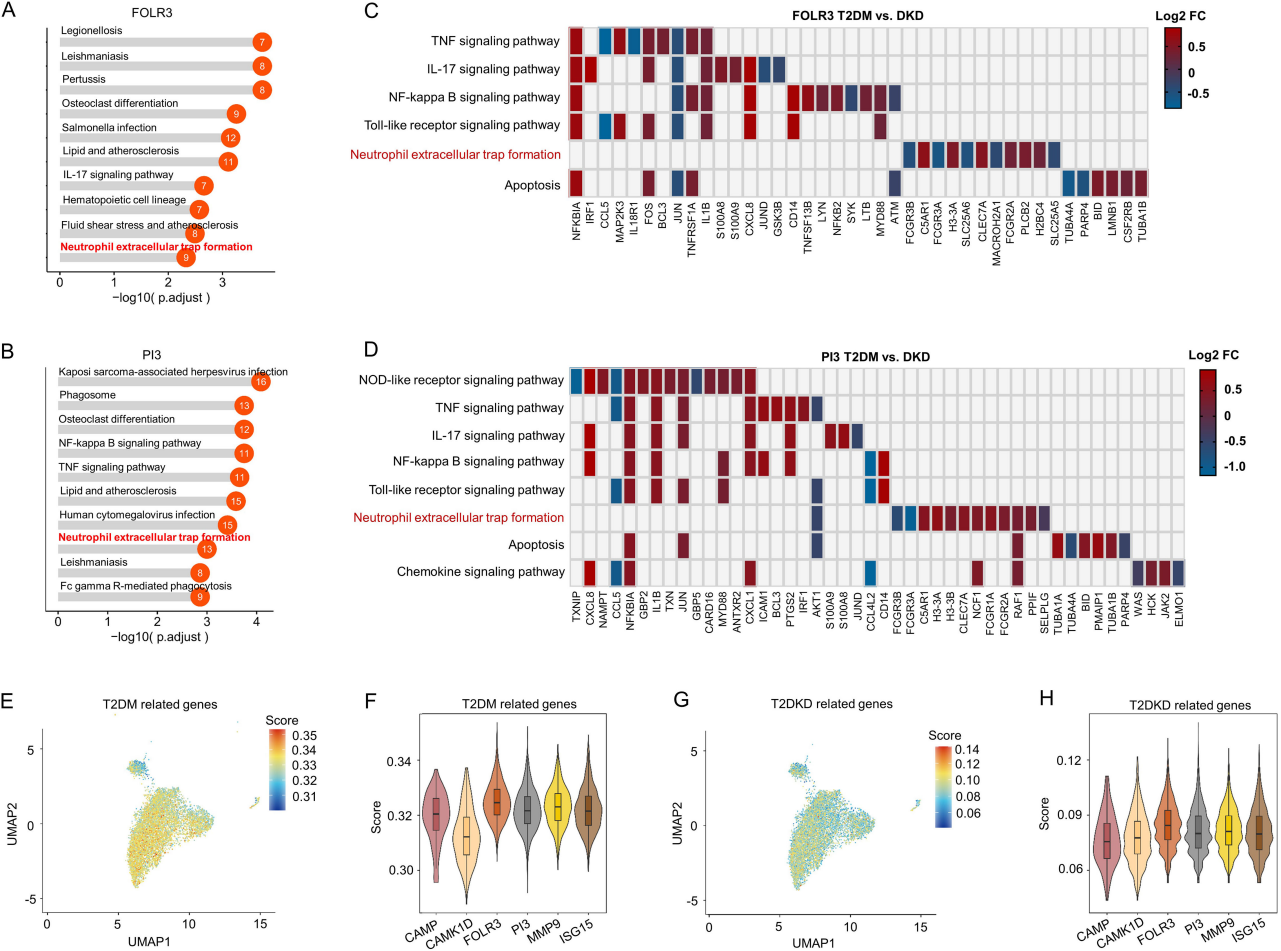
Functional and gene expression analysis of neutrophil sub-cell clusters. **(A, B)** Functional analysis of FOLR3 **(A)** and PI3 **(B)** sub-cell clusters of neutrophils using KEGG enrichment analysis. **(C, D)** Differences in gene expression within immune-related KEGG pathways, shown by Log 2FC, between patients with T2DM and DKD in FOLR3 **(C)** and PI3 **(D)** clusters. **(E, F)** UMAP dimension reduction **(E)** and violin plots **(F)** illustrating T2DM-related gene set scores across neutrophil sub-cell clusters. **(G, H)** UMAP dimension reduction **(G)** and violin plots **(H)** illustrating DKD-related gene set scores across neutrophil sub-cell clusters.

Functional analysis of these neutrophil sub-cell clusters indicated enrichment of the NET pathway within the FOLR3 and PI3 clusters, which were the most prevalent in patients with T2DM and T2DKD (**Figures 3A, B**). This finding implies that NETs may contribute to DKD progression. However, the specific molecular constituents enriched in the NET pathway varied between FOLR3 and PI3 clusters (**Figures 3C, D**).

To ascertain the associations between neutrophil sub-cell clusters and T2DM/T2DKD, functional associations between neutrophil sub-clusters and T2DM/T2DKD were evaluated using disease-specific gene sets curated from GeneCards (relevance score >5). The analysis revealed that, despite some variability in individual gene scores, the FOLR3 and PI3 subpopulations consistently showed higher cumulative scores for both T2DM and T2DKD gene sets compared to other subpopulations (**Figures 3E–H**). Specifically, while genes such as CAMP and CAMK1D in certain subpopulations might have lower individual scores in some plots, the overall scoring system takes into account the combined contribution of multiple genes within each gene set. Although absolute UCell scores were modest (ranging from 0.04 to 0.35), relative comparisons revealed consistent patterns. For T2DM gene sets, FOLR3 and PI3 subclusters showed 1.02- to 1.10-fold higher scores than other subclusters (**Supplementary Table 4**). For T2DKD gene sets, FOLR3 and PI3 exhibited 1.00- to 1.03-fold enrichment over other subclusters (**Supplementary Table 4**). This directional consistency, rather than absolute score magnitude, highlights their potential disease relevance.

### 4.4 *In vitro* validation of enhanced NET formation in neutrophils exposed to high glucose and T2DKD serum

Building on scLacNAc-seq findings, which indicated an increase in neutrophil subpopulations associated with NET formation in patients with T2DM and T2DKD, potentially linked to disease progression, we conducted an *in vitro* study to validate these findings. Fresh blood samples were obtained from 10 healthy volunteers and neutrophils were isolated and cultured under three conditions for 30 h, respectively: normal medium (control), medium supplemented with 30 mM glucose (HG), and 30 mM high-glucose medium + 10% serum pooled from T2DKD patients (HG + DKD serum). Subsequently, a subset of these cells was assessed for viability using the CCK8 assay, whereas the supernatants were reserved for ELISA analysis and cell lysates for WB detection.

**Figure 4:**
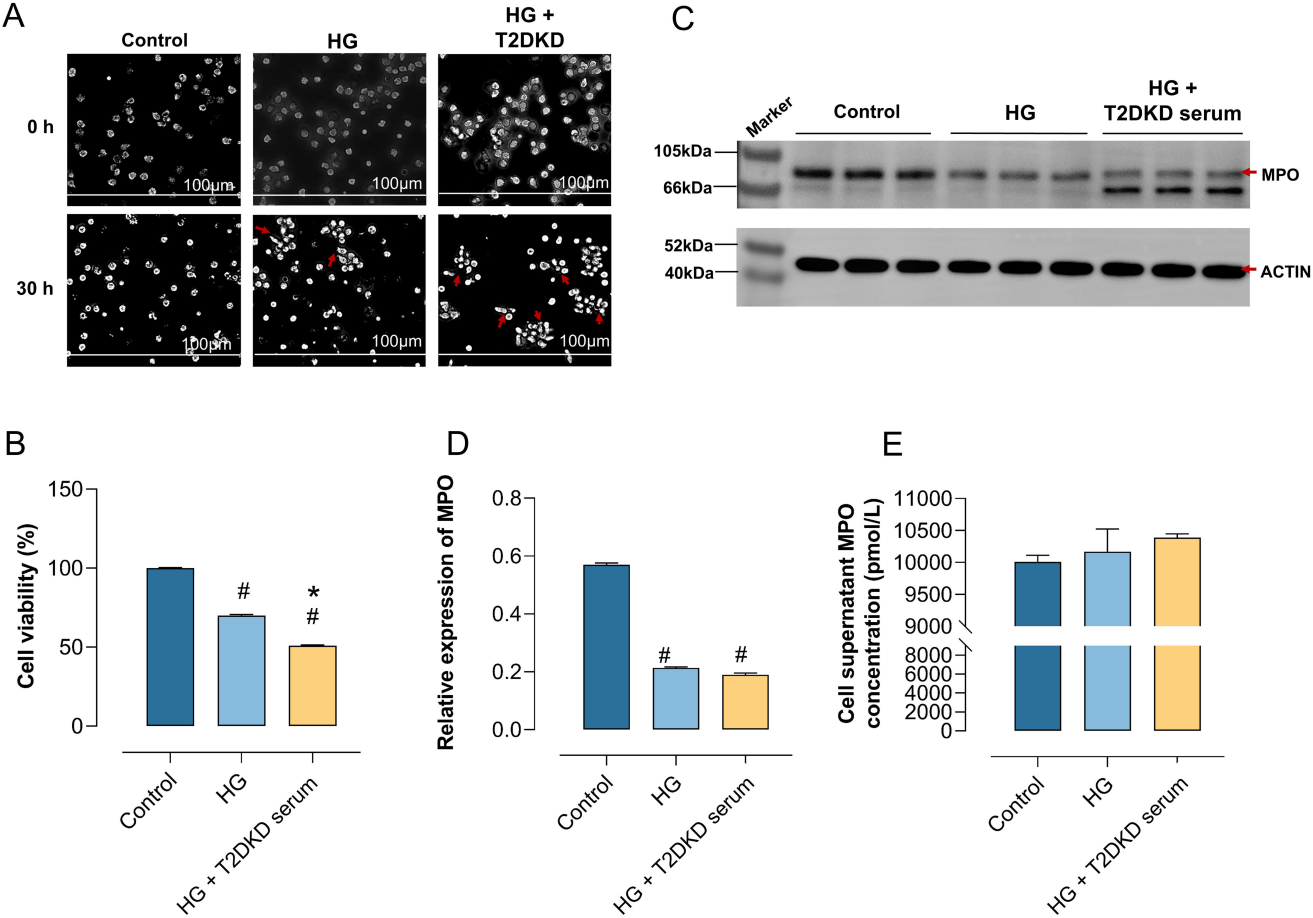
Enhanced NET formation in neutrophils under DKD-Like conditions. **(A)** Human primary neutrophils were cultured in normal medium (control), high glucose medium (30 mmol/L) (HG), and high glucose with DKD patient serum (HG + DKD serum). Neutrophil changes were observed microscopically (40x) at 0h and 30h. **(B)** CCK8 assay assessed the activity of neutrophils under various *in vitro* culture conditions. **(C, D)** WB analysis examined MPO protein expression, a marker of NET formation, in neutrophils under different conditions **(C)**, with semi-quantitative analysis results **(D)**. **(E)** ELISA measured MPO concentration in the supernatant of neutrophils under different culture conditions. # indicates a statistically significant difference between the HG group and HG+T2DKD serum groupcompared to the control group (P < 0.05). * indicates a statistically significant difference between HG+T2DKD serum group and the HG group.

The findings revealed that, compared to the control group, neutrophils exposed to HG and HG + T2DKD serum exhibited a significant increase in cells displaying morphological alterations suggestive of cell distress or death, including rupture (**Figure 4A**), accompanied by a marked decrease in overall cell viability (**Figure 4B**). Moreover, comparing to HG group, HG + T2DKD serum group exhibited a further reduction in cell viability. Although, morphological changes are non-specific but consistent with NET-associated stress.

Notably, WB analysis indicated a downregulation of intracellular MPO expression at protein level in neutrophils cultured under HG and HG + T2DKD serum conditions relative to the control (**Figures 4C, D**). Conversely, ELISA results demonstrated a significant upregulation of MPO levels in the corresponding supernatants (**Figure 4E**). This pattern of reduced intracellular MPO coupled with increased extracellular MPO release is a recognized hallmark of NET formation.

Collectively, these data suggest that the conditions mimicking hyperglycemia and the serum environment of patients with T2DKD promote key features associated with NET formation in neutrophils, as evidenced by the characteristic MPO release pattern. The observed morphological changes and reduced cell viability are consistent with the cellular stress and lytic processes involved in NET formation. Elevated levels of neutrophil chemokines, receptors, and NET formation markers in plasma of patients with T2DM and T2DKD.

### 4.5 Elevated levels of neutrophil chemokines, receptors, and NETosis markers in plasma of patients with T2DM and T2DKD

**Figure 5:**
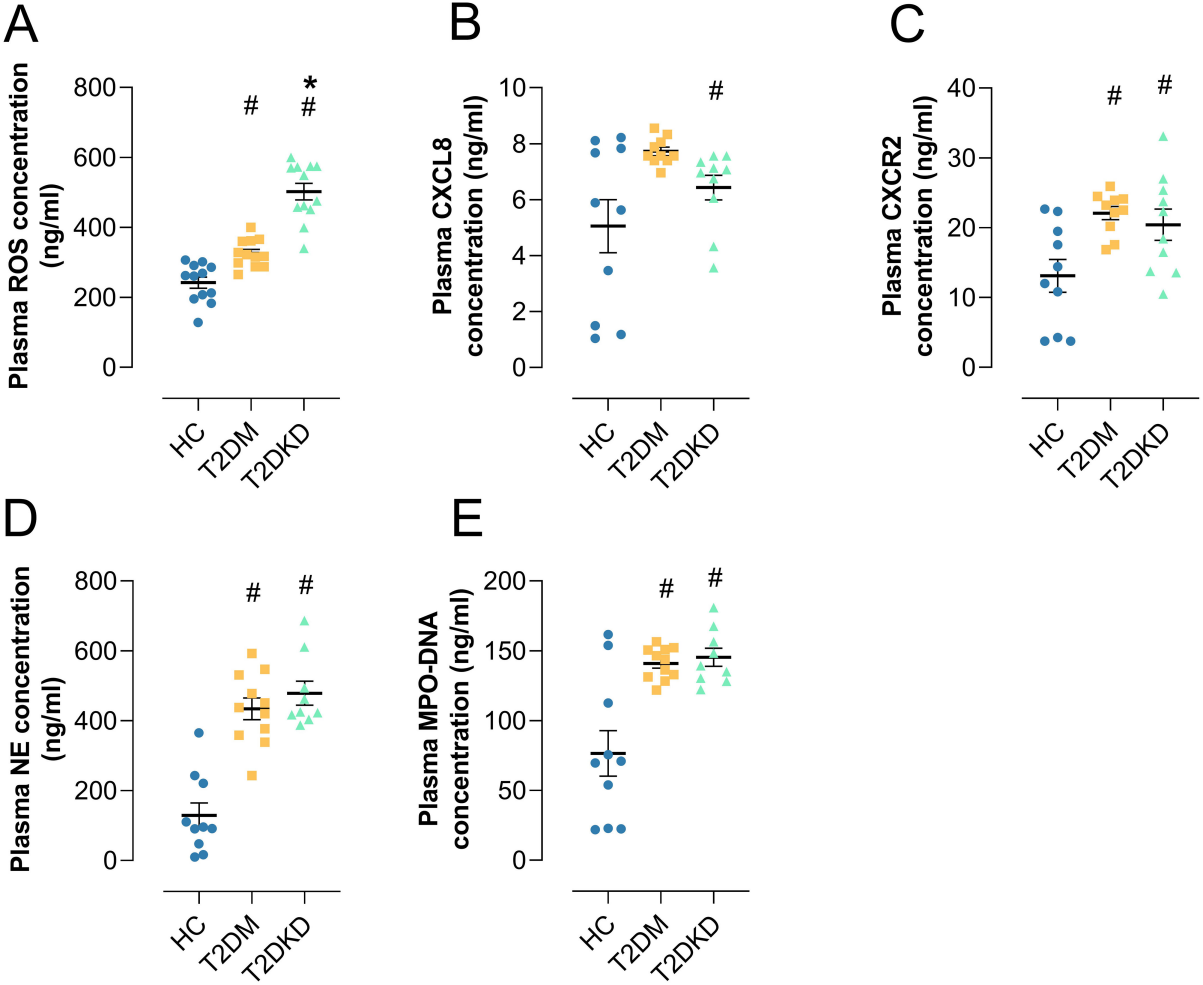
Elevated plasma biomarkers in patients with T2DM and T2DKD. **(A)** Plasma level of ROS. **(B, C)** Plasma levels of neutrophil chemokine CXCL8 **(B)** and its receptor CXCR2 **(C)**. **(D, E)** Plasma levels of NET formation markers: NE **(D)** and MPO **(E)**. # indicates a statistically significant difference between the T2DM group and T2DKD group compared to the HC group (P < 0.05). * indicates a statistically significant difference between T2DKD group and the T2DM group.

We conducted a series of ELISA analyses on plasma samples from patients with T2DM and T2DKD. Compared to HCs, plasma levels of the oxidative stress marker ROS were significantly elevated in both T2DM and T2DKD patients (**Figure 5A**), with T2DKD patients exhibiting a more pronounced increase than T2DM patients (**Figure 5A**). Furthermore, the plasma concentrations of the neutrophil chemokine CXCL8 and its receptor CXCR2 were markedly elevated in patients with T2DM and T2DKD compared to those in HCs (**Figures 5B, C**). Similarly, the levels of NET formation markers, including NE and MPO-DNA, were significantly higher in patients with T2DM and T2DKD than in the HCs (**Figures 5E, F**).

While plasma levels of NET biomarkers (MPO-DNA, NE) and ROS were elevated in patients with T2DM and T2DKD, Pearson correlation analysis showed no significant correlation between individual NET markers and ROS levels (**Supplementary Figure 4**). This may be attributed to the limited sample size (n=20 per group) and the complexity of NET formation pathways beyond ROS dependency. Future large-scale studies will explicitly examine this relationship. Separately, ROS levels showed significant positive correlation with NLR specifically in T2DKD patients (*r*=0.73, *P*=0.02), but not in HCs (*r*=0.36, *P*=0.63) or T2DM (*r*=-0.04, *P*=0.29) (**Supplementary Figure 4**). This suggests compartmentalized relationships between oxidative stress and systemic inflammation in T2DKD (30).

### 4.6 Enhanced interactions between neutrophils and MPs through CXCL8 and CXCR2 in patients with T2DM and T2DKD compared to HC

Using established markers, we identified MP sub-cell clusters including classical mononuclear cells (ClassicalMono), non-classical monocytes (NonClassicalMono), conventional dendritic cells (cDCs), and plasmacytoid dendritic cells (pDCs) (**Supplementary Table 3**). Notably, the prevalence and proportion of ClassicalMono were significantly reduced in patients with T2DM and T2DKD compared to those in HCs (**Supplementary Table 3**).

Given that neutrophils undergoing senescence and NET formation are typically cleared by MPs (31), the scLacNAc-seq results, which showed a significant increase in FOLR3 and PI3 subpopulations associated with aging and NET formation in patients with T2DM and T2DKD, prompted us to hypothesize enhanced neutrophil-MP interactions.

We assessed the aging characteristics of each neutrophil sub-cluster by UCell scoring for established senescence-associated gene sets (Hallmark Senescence from the GSEA website). The FOLR3 and PI3 subclusters consistently exhibited the highest senescence scores (**Supplementary Figure 5B**), indicative of the most pronounced aging features. Concurrently, we evaluated the stemness/differentiation potential of each cluster by analyzing the entropy of gene sets associated with cell proliferation and differentiation. The CAMP cluster displayed the highest stemness (lowest entropy) and lowest senescence scores, suggesting a more progenitor-like state. In contrast, the FOLR3 and PI3 subclusters showed significantly lower stemness (higher entropy) and the highest senescence scores (**Supplementary Figures 5A, B**), consistent with a more differentiated and aged phenotype.

Pseudotime analysis was performed to infer a potential differentiation trajectory. While the inferred trajectory suggests a progression (**Supplementary Figure 5C**), it is important to note that the FOLR3 and PI3 subclusters, which bear the strongest senescence signatures, occupy intermediate positions along this trajectory rather than its terminus. Their intermediate position, coupled with their high senescence scores and low stemness, supports their identification as neutrophil subsets exhibiting prominent aging characteristics.

**Figure 6:**
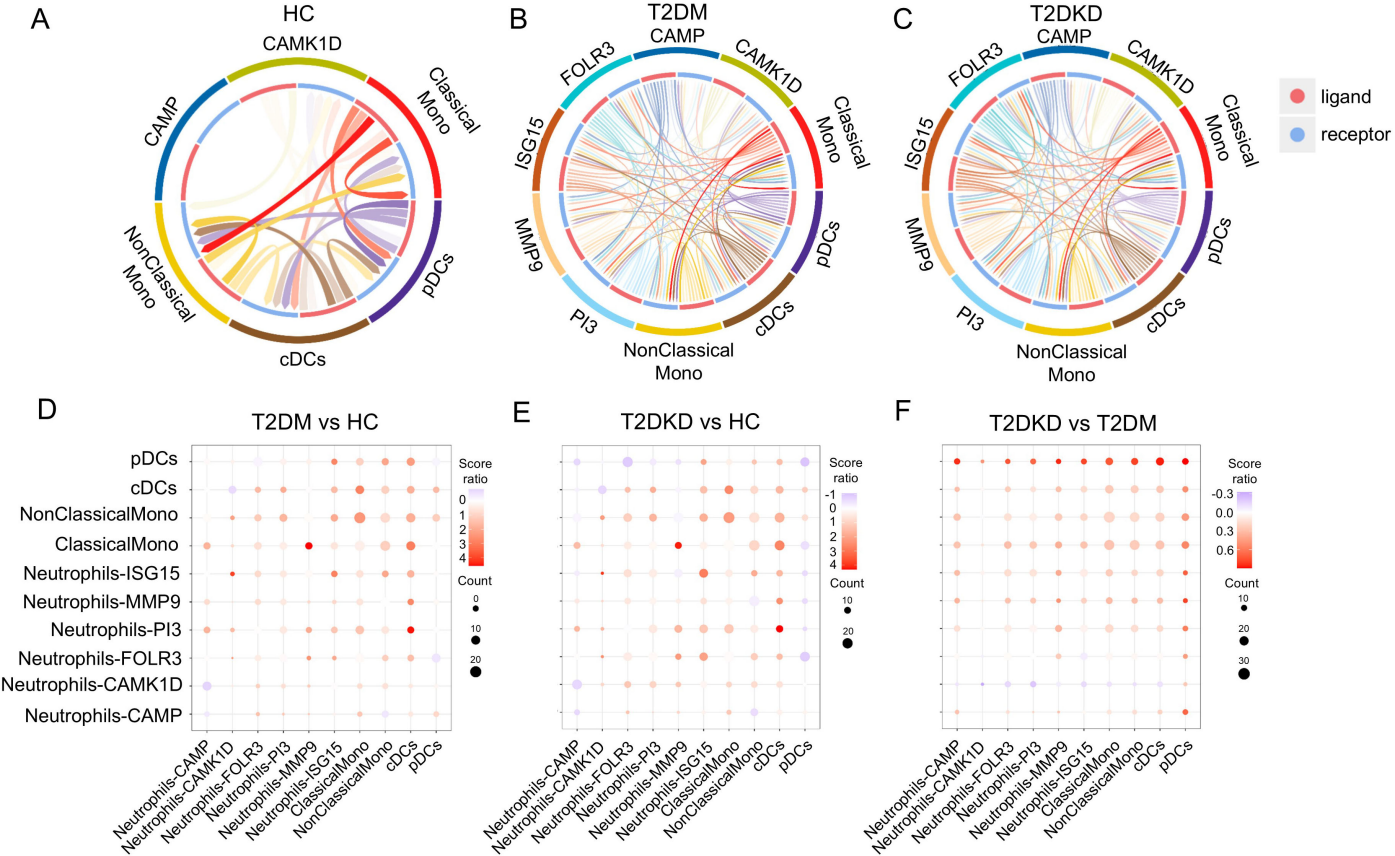
Enhanced cell-cell interactions in patients with T2DM and DKD. **(A–C)** Chord diagrams illustrating interactions between MP and neutrophil sub-cell clusters in HC **(A)**, patients with T2DM **(B)**, and patients with T2DKD **(C)**. **(D–F)** Bubble plots depicting changes in interaction intensity between ligand and receptor cell clusters: T2DM *vs*. HC **(D)**, T2DKD *vs*. HC **(E)**, and T2DKD *vs*. T2DM **(F)**.

Cell-cell interaction analysis among six neutrophil sub-cell clusters and four MP sub-cell clusters using the CellPhoneDB method corroborated our hypothesis. In HCs, minimal interactions were noted between neutrophil sub-cell clusters (CAMP and CAMK1D) and MP sub-cell clusters (ClassicalMono, NonClassicalMono, cDCs, pDCs) (**Figure 6A**). In contrast, patients with T2DM and T2DKD displayed numerous and more intense interactions, particularly between neutrophil sub-cell clusters (CAMK1D, CAMP, FOLR3, ISG15, MMP9, PI3) and MP sub-cell clusters (ClassicalMono, NonClassicalMono, cDCs, and pDCs) (**Figures 6B–E**). The interaction intensity was particularly elevated in T2DKD patients compared with that in T2DM patients (**Figure 6F**).

The interactions between MPs and neutrophils in T2DM and T2DKD patients were found to be predominantly mediated by CXCL8 and CXCR2 ligand-receptor pairs, with the strongest and most significant interactions observed between ClassicalMono MPs and FOLR3 and PI3 neutrophil sub-clusters (**Supplementary Figure 6**). Further analysis revealed upregulated expression of CXCL8 and CXCR2 in MPs and neutrophils in T2DM and T2DKD patients compared to that in HCs, with neutrophils showing higher expression levels (**Supplementary Figures 7A–D**).

## 5 Discussion

In this study, advanced single-cell sequencing (scLacNAc-seq) and plasma biomarker analyses were employed to comprehensively characterize the clinical and immunological profiles of patients with T2DM and T2DKD. Our findings uncovered significant alterations in immune cell populations, particularly LDGs and monocyte subsets, and underscored the potential role of dysregulated NET formation in the progression of T2DKD. These insights contribute to a more profound understanding of the immunological mechanisms underlying diabetic complications and may inform the development of future therapeutic strategies.

Although neutrophil counts did not differ significantly among groups, the NLR was markedly elevated in T2DKD patients. As a recognized marker of systemic inflammation with prognostic value in chronic diseases, including diabetes (32, 33), the increased NLR supports its relevance as an indicator of inflammation and disease severity in DKD (32, 33).

The scLacNAc-seq analysis revealed an expansion of LDGs with elevated terminal N-acetylglucosamine (GlcNAc) exposure in T2DKD patients. This glycosignature is associated with pathogenic neutrophil activation (33, 34). The elevated glycosylation levels in these neutrophils suggest enhanced post-translational modifications, which may influence neutrophil functions such as adhesion to endothelial cells, cell-cell interactions, and NET formation (23, 34). Notably, disease-enriched LDG subpopulations (FOLR3 and PI3) showed co-enrichment of GlcNAc termini and NET formation pathways, indicating glycosylation-mediated dysregulation of neutrophil death programs (31, 34, 35). This finding aligns with previous reports that hyperglycemia promotes vital NET release through mitochondrial ROS- PAD4 activation (9).

Hyperglycemia reprograms neutrophil transcriptomes towards a proinflammatory state, increasing their sensitivity to NET-inducing stimuli (36, 37). In our study, the upregulation of the CXCL8/CXCR2 axis in LDGs was observed. This axis primes neutrophils for NET release under metabolic stress (37, 38). Transcriptomic studies have also shown that hyperglycemia-induced changes in gene expression, related to inflammation and lipid metabolism, contribute to neutrophil dysregulation in T2DM (39). Neutrophils from diabetic patients exhibit enhanced basal NET formation and a heightened response to stimulation, associated with metabolic reprogramming that promotes histone acetylation essential for NET priming (37).

Functionally, our *in vitro* study, simulating the T2DKD environment, confirmed that hyperglycemia directly triggers lytic NET release. Reduced intracellular MPO and elevated extracellular MPO-DNA complexes, consistent with ROS-driven NADPH oxidase activation in high-glucose conditions, supported this conclusion. ROS, primarily generated under high glucose, not only cause oxidative stress and cellular damage but also act as significant inducers of NET formation (9, 40–42). Elevated plasma ROS levels in T2DM and T2DKD patients, coupled with a positive correlation between ROS levels and NLR, highlight a synergistic relationship exacerbating neutrophil recruitment (40, 41), inflammatory signaling, and kidney damage (41, 43).

Through NET formation, neutrophils release numerous proteases that serve a dual function in host defense and potential tissue damage (34). As reported by Zheng et al., NETs contribute to the progression of DKD by triggering pyroptosis in glomerular endothelial cells, as evidenced by both *in vivo* and *in vitro* DKD models (44). Their research further demonstrated that the degradation of NETs’ double-stranded DNA by DNase I significantly ameliorated streptozotocin-induced glomerular endothelial cell damage in a type 1 DKD mouse model (44). The results of the current study, employing scLacNAc-seq, are in concordance with these findings, implicating NET formation in the progression of DKD (15, 30) and offering clinical evidence to support this association.

Beyond the changes in neutrophils, T2DM and T2DKD patients showed a reduction in ClassicalMono and an increase in non-classical monocyte subsets, indicating a shift towards more proinflammatory phenotypes. The analysis of cell-cell interactions revealed intensified engagement between specific neutrophil subpopulations (FOLR3 and PI3 LDGs) and ClassicalMono, potentially modulated by GlcNAc-modified surface ligands (23). Mediated predominantly by the CXCL8/CXCR2 axis, these enhanced interactions may facilitate the clearance of NET-forming neutrophils by MPs (45), exacerbating and perpetuating inflammatory responses and hindering inflammation resolution, thus contributing to chronic tissue injury (46). Targeting the CXCL8/CXCR2 signaling pathway emerges as a promising therapeutic strategy to mitigate DKD progression (47).

Our data establish ROS overproduction as a potential link between hyperglycemia, NET dysregulation, and renal damage. Therefore, therapeutic strategies aimed at reducing oxidative stress may offer dual benefits by attenuating both oxidative and inflammatory pathways in T2DKD (48). While conventional antioxidants have shown limited efficacy in clinical T2DKD trials (49, 50), mitochondria-targeted agents offer promise (10, 51). For example, MitoQ suppresses hyperglycemia-induced mitochondrial ROS, reducing NET formation and glomerular injury in diabetic mice (10). Sodium-glucose cotransporter 2 (SGLT2) inhibitors, such as empagliflozin, indirectly lower oxidative stress by improving glycemia and mitochondrial function, correlating with reduced NLR and proteinuria in patients (52, 53). Combining NET inhibitors (e.g., anti-CXCL8) with mitochondria stabilizers may synergistically attenuate NET-driven inflammation and tissue damage.

Collectively, our data implicate GlcNAc-high LDGs as central drivers of NETs-mediated injury in T2DKD. Targeting glycosylation-dependent neutrophil priming (e.g., via ROS/PAD4 inhibition) or disrupting CXCL8/CXCR2 signaling (47) represents a promising therapeutic strategy to mitigate renal damage.

While our study provides valuable insights into immunological alterations in T2DM and T2DKD, it has certain limitations. The cross-sectional design restricts the ability to establish causal relationships between the observed immune changes and disease progression. Longitudinal studies are necessary to delineate the temporal dynamics of neutrophil and MP alterations in diabetes. Second, renal infiltrating LDGs were not profiled, which limits the understanding of their direct role in kidney pathology. Third, further functional assays are required to elucidate the mechanistic roles of identified neutrophil subpopulations and their interactions with monocytes. Fourth, NET formation was mainly assessed by MPO release, a validated biochemical marker. However, future work should include morphological quantification, such as measuring the nuclear area and the percentage of NET-positive cells, to enhance the rigor of the research.

## 6 Conclusion

**Figure 7:**
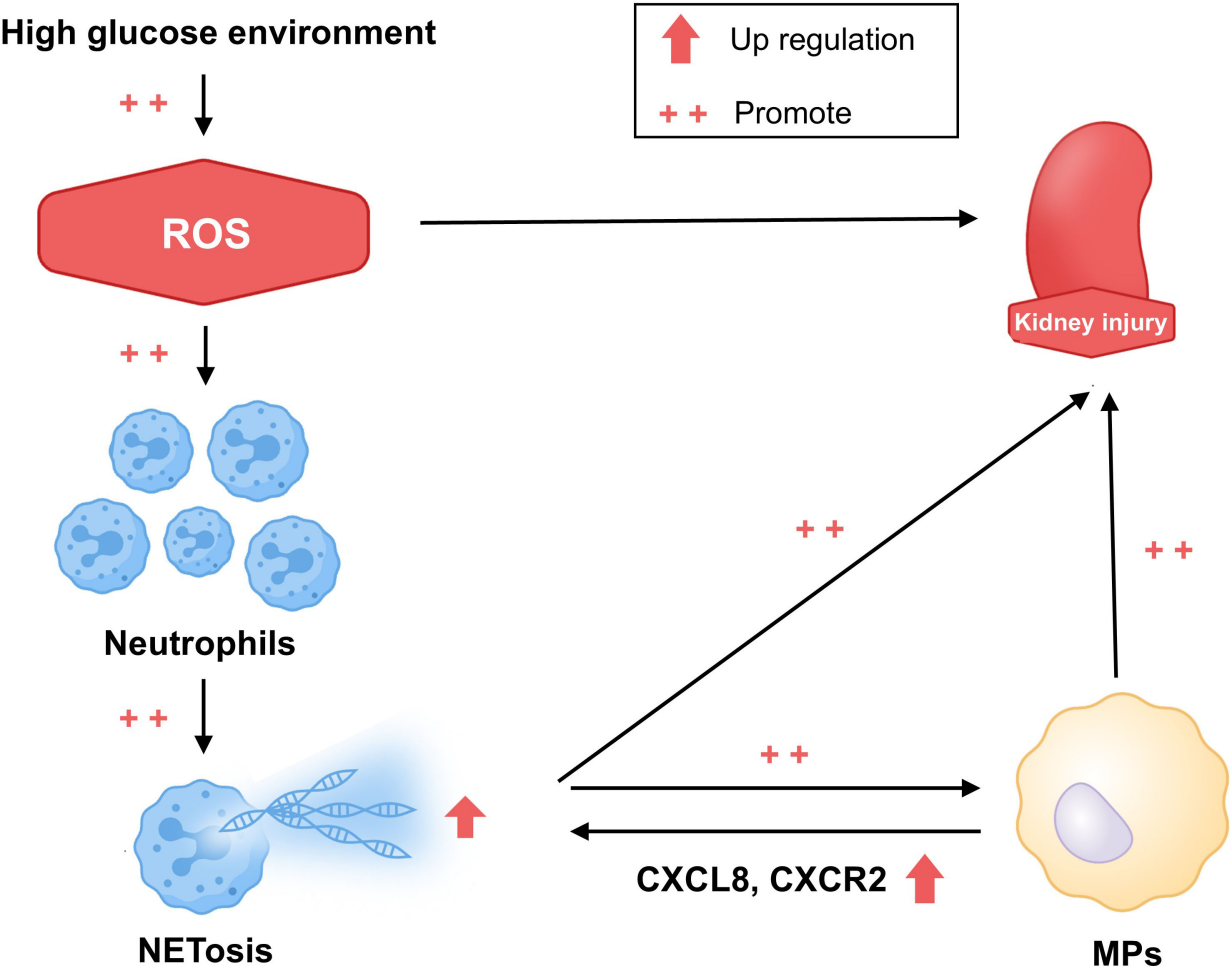
Hypothesized mechanism of the progression of kidney damage in patients with T2DM and T2DKD. This schematic diagram illustrates the proposed hypothesis. In patients with T2DM and T2DKD, prolonged hyperglycemia results in elevated ROS levels, promoting kidney damage. This triggers inflammatory responses, leading to neutrophil aggregation and increased NET formation, which subsequently activate MPs through CXCL8 and CXCR2 interactions. Altered neutrophil and MP levels and functions further intensify inflammation, exacerbating kidney damage.

This study documents immunological alterations in patients with T2DM and T2DKD, including neutrophil expansion, oxidative stress-associated NET formation enhancement, and CXCL8/CXCR2-mediated changes in interactions with MPs (**Figure 7**). The results indicate a role of neutrophil-driven inflammation in T2DKD progression. Targeting these immune pathways may represent potential therapeutic approaches for preventing or slowing kidney disease progression in diabetic patients.

## Data Availability

The original contributions presented in the study are included in the article/**Supplementary Material**, further inquiries can be directed to the corresponding author/s.
