# Supplementary material for: Immunological profiling in type 2 diabetes mellitus and type 2 diabetic kidney disease: insights from single-cell LacNAc sequencing
Source: Front Endocrinol (Lausanne). 2025 Aug 11;16:1550925. doi: 10.3389/fendo.2025.1550925 (PMC12375462; doi:10.3389/fendo.2025.1550925)
Supplement: Supplementary file 1 [file DataSheet1.docx]

Supplementary Material

# Supplementary Figures and Tables

## Supplementary Figures


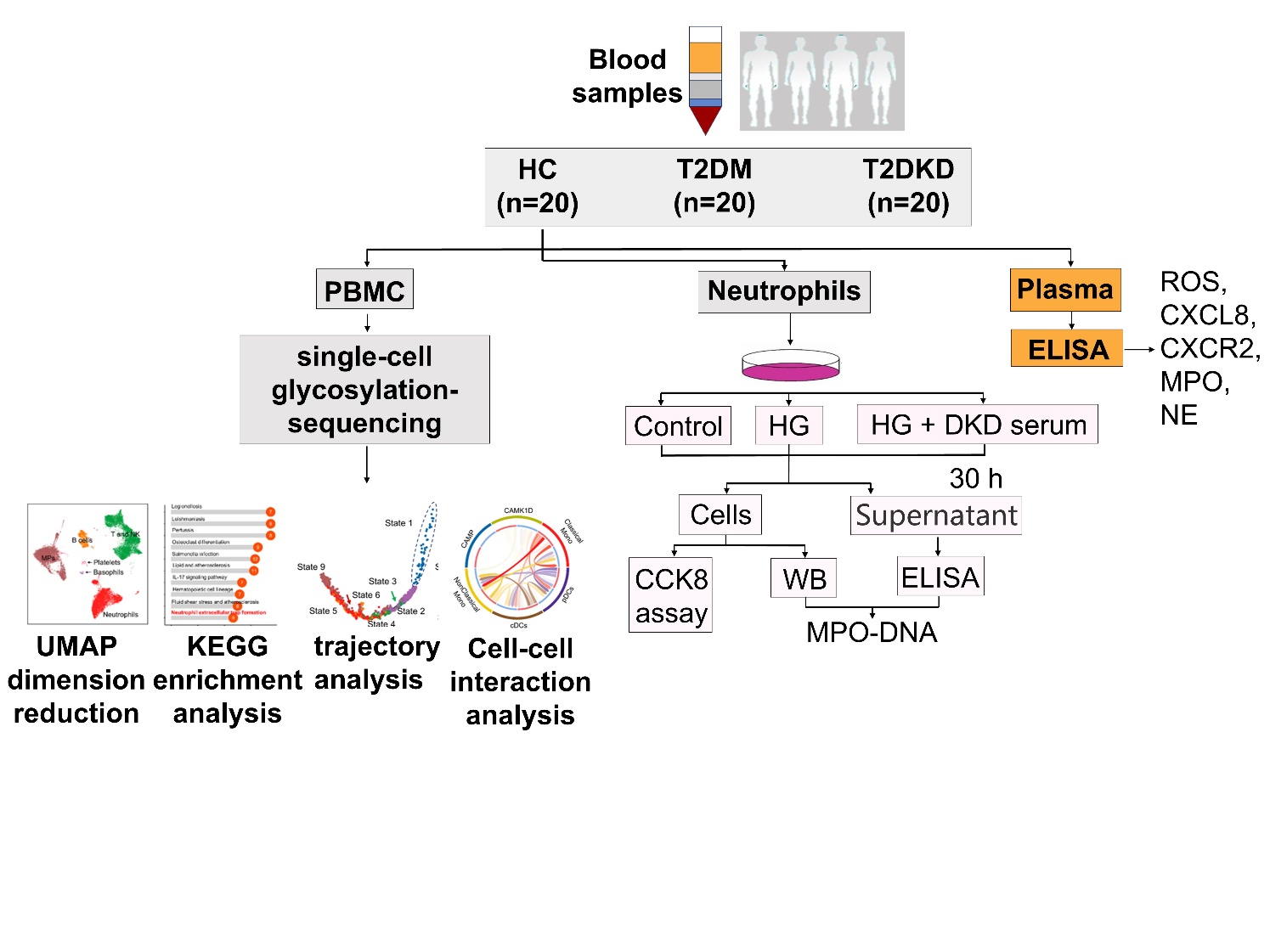


**Supplementary Figure 1. Flowchart of the study design**

Twenty participants were recruited for each group: HC, patients with T2DM, and patients with T2DKD. Blood samples were collected from all participants. PBMCs were isolated from 10 samples per group for scLacNAc-seq and subsequent downstream analyses. Additionally, neutrophils were isolated from 10 healthy individuals for in vitro studies. Cells and supernatants under different culture conditions (including normal, HG, and HG plus T2DKD serum) were harvested after 30 hours. MPO expression was assessed in the cells and supernatants using WB and ELISA, respectively. Serum samples were separated from all blood samples to measure the concentrations of ROS, CXCL8, CXCR2, MPO, and NE via ELISA.

**
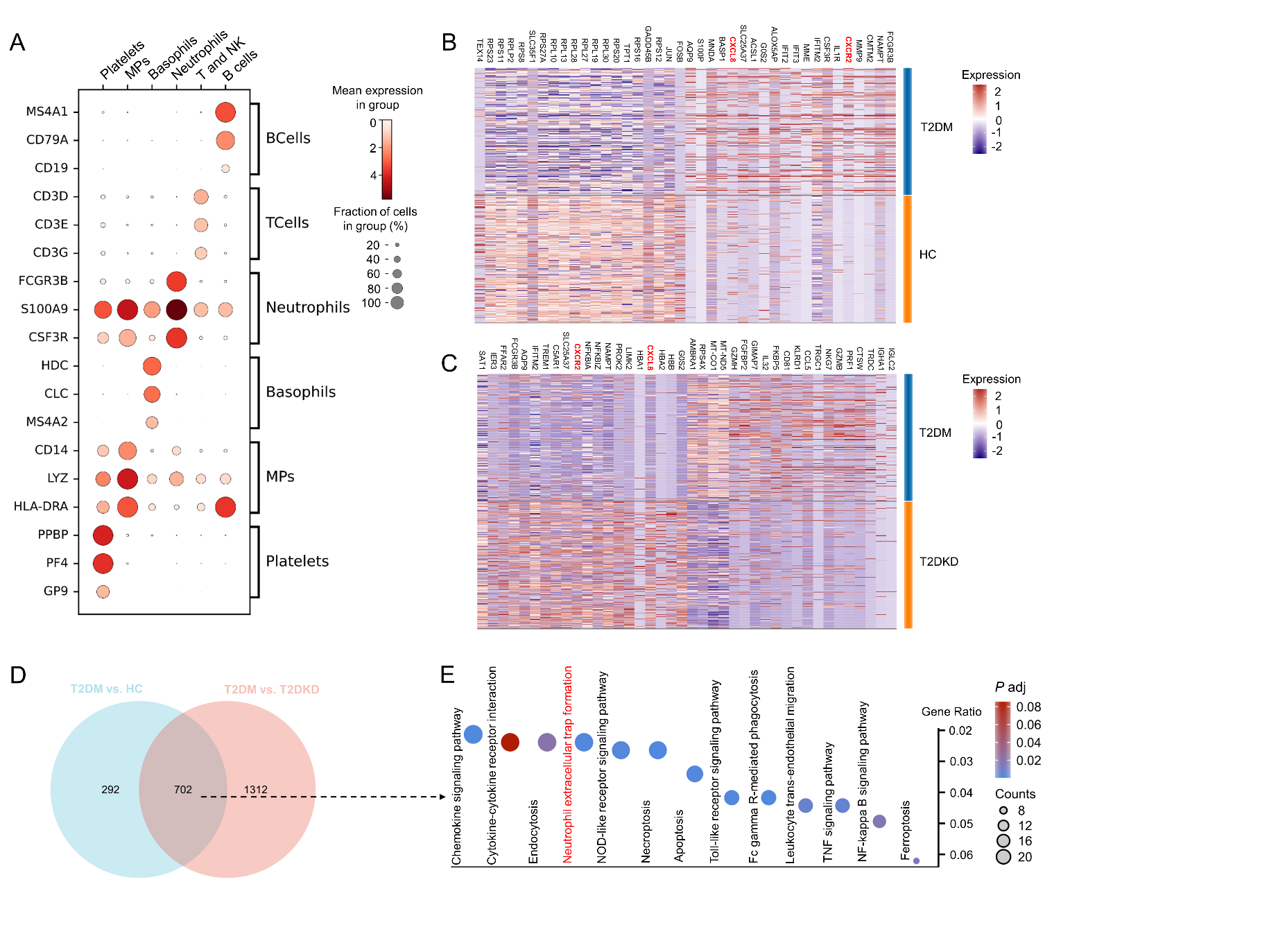
**

**Supplementary Figure 2. Comparative transcriptomic analysis of PBMCs in HC, and diabetic patients**

A. Relative expression levels of marker genes across different cell clusters.

B-C. Heatmaps displaying the expression of DEGs in PBMCs between: T2DM patients and HC (B), as well as between T2DM patients and T2DKD patients (C).

D. Venn diagram illustrating the 702 overlapping DEGs identified in both comparisons: HC vs. T2DM and T2DM vs. T2DKD.

E. Top KEGG pathways enriched among the 702 overlapping DEGs.


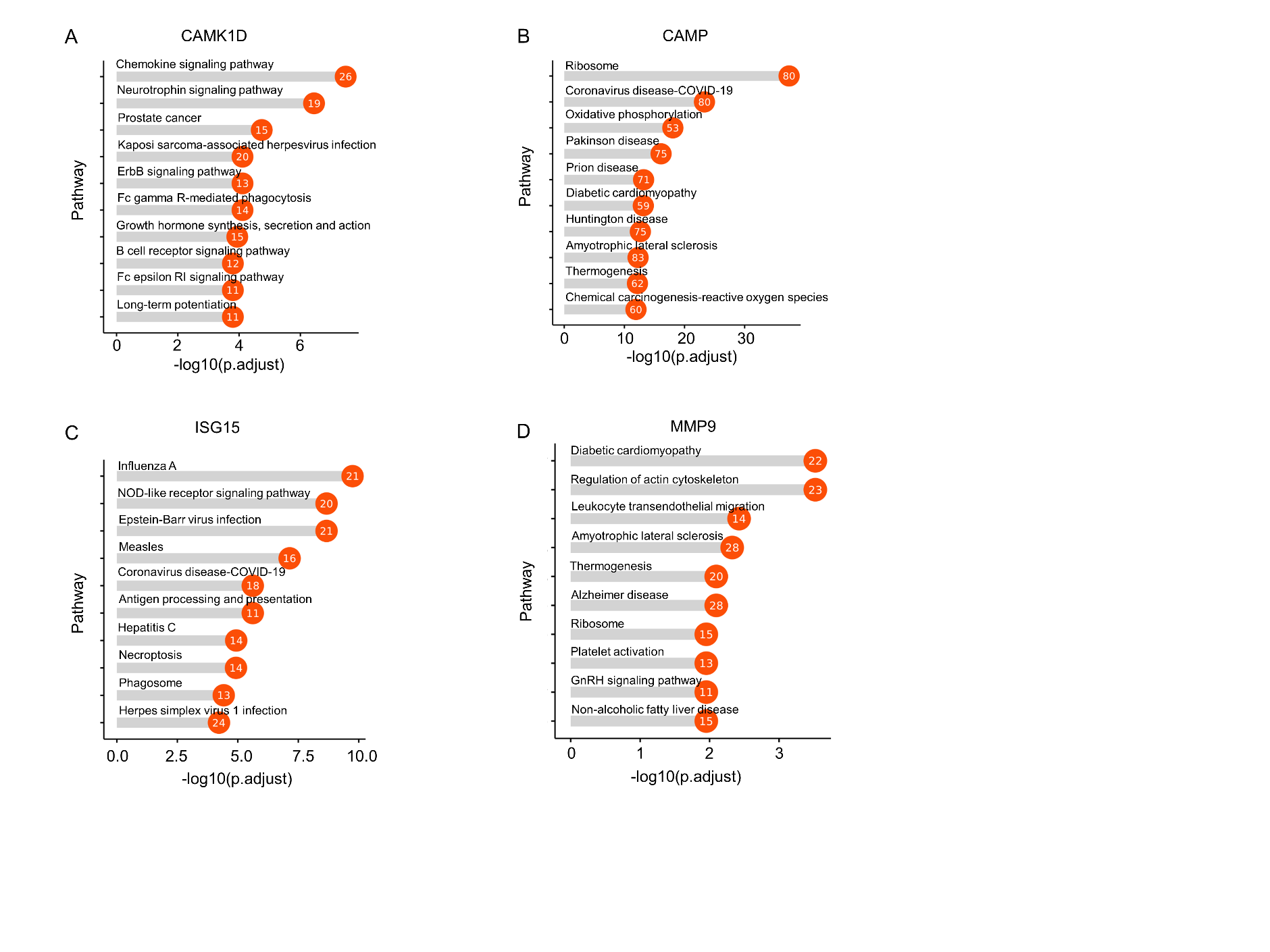


**Supplementary Figure 3. KEGG pathway enrichment analysis of neutrophil sub-clusters**

A-D. KEGG pathway enrichment analyses for the following neutrophil sub-cell clusters: CAMK1D (A), CAMP (B), ISG15 (C), MMP9 (D).

**
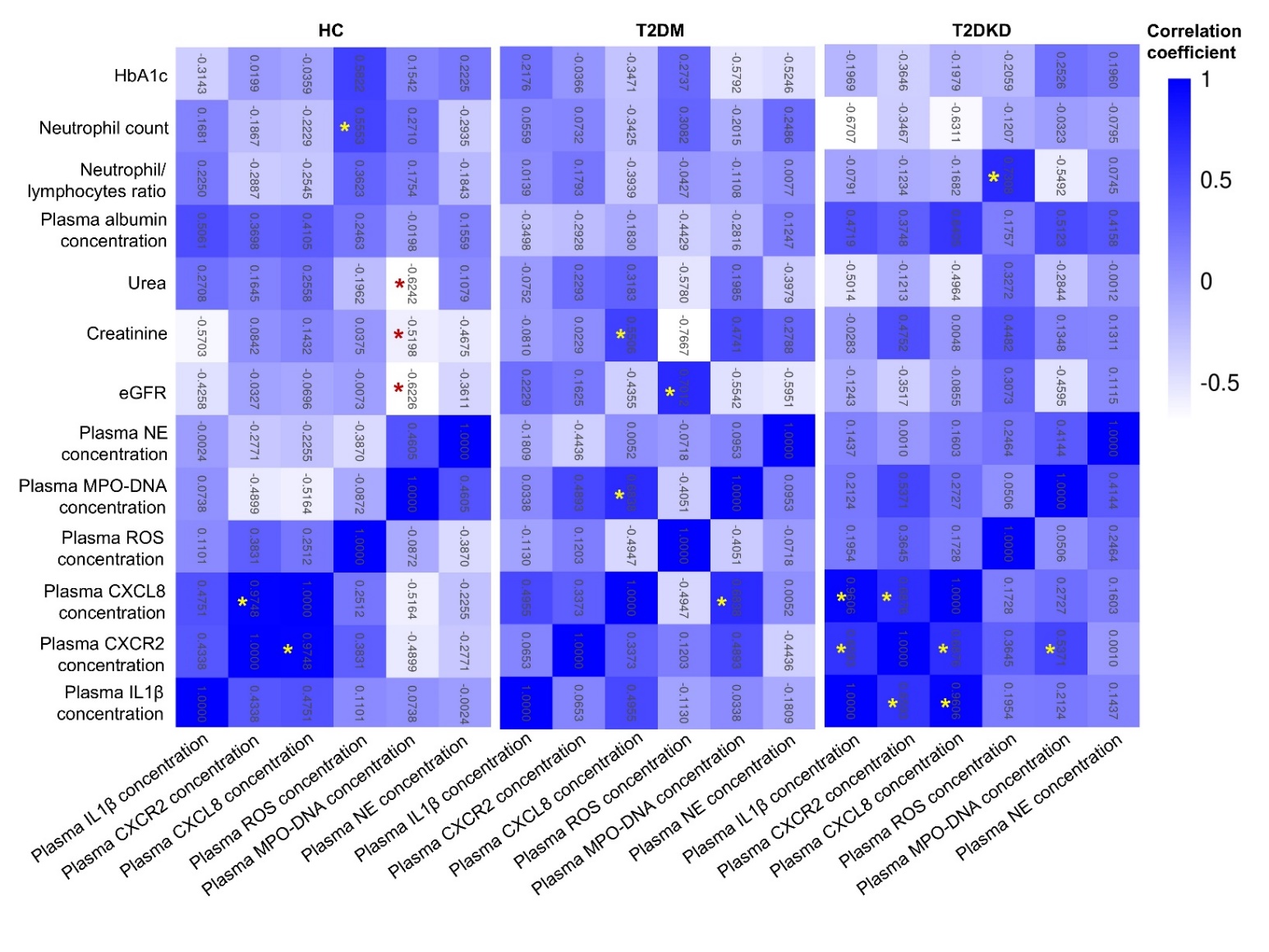
**

**Supplementary Figure 4. Correlation between plasma ROS levels and neutrophil/lymphocyte ratio across groups**

Scatter plots illustrating the relationship between plasma ROS concentrations and the neutrophil-to-lymphocyte ratio in HCs (left), patients with T2DM (middle), and patients with T2DKD (right). Pearson correlation analysis was performed for each group.

**Note:** * indicates a statistically significant Pearson correlation coefficient (*P* < 0.05).


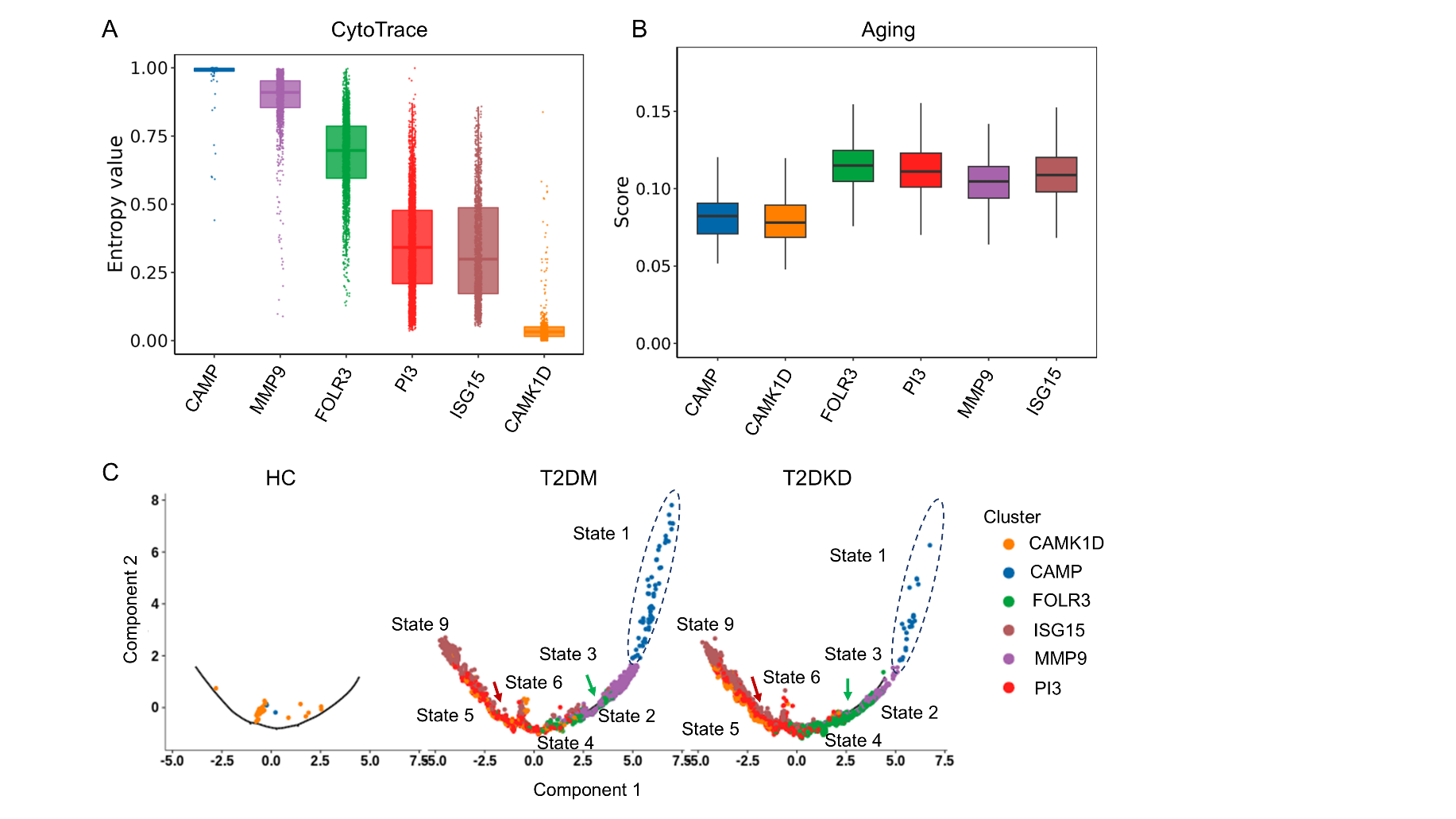


**Supplementary Figure 5. Gene set scoring and pseudotime analysis of neutrophil subclusters**

A. Assessment of cellular stemness in each neutrophil subcluster was performed by analyzing the entropy of gene sets associated with cell proliferation and differentiation.

B. Evaluation of aging in each neutrophil subcluster was conducted by scoring gene sets related to aging processes.

C. Cell differentiation trajectories were analyzed using pseudotime analysis for each neutrophil subcluster.


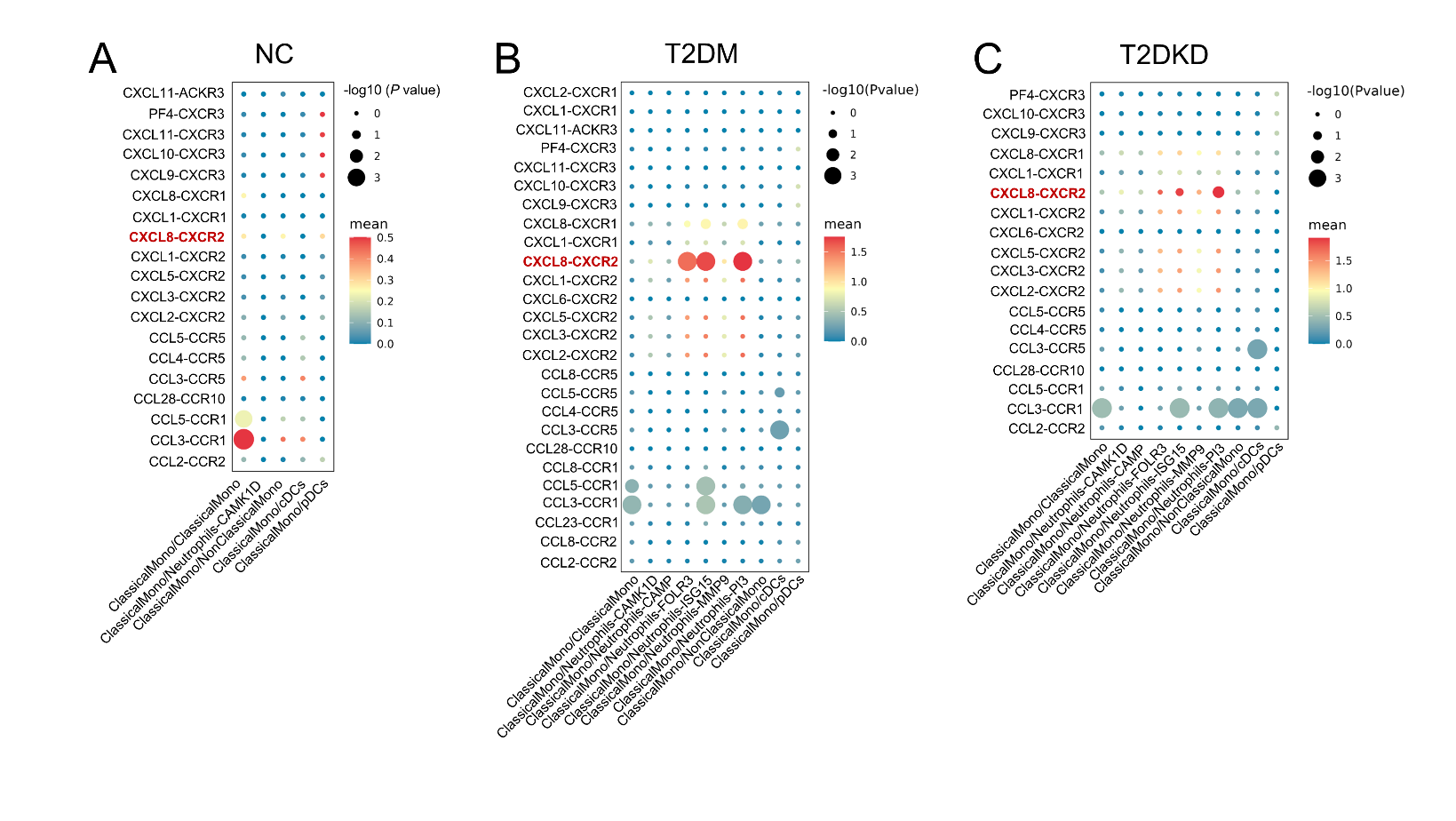


**Supplementary Figure 6. Ligand–receptor pairs mediating cell-cell interactions between MP and neutrophil sub-clusters in HC (A), patients with T2DM (B), and patients with T2DKD (C)**


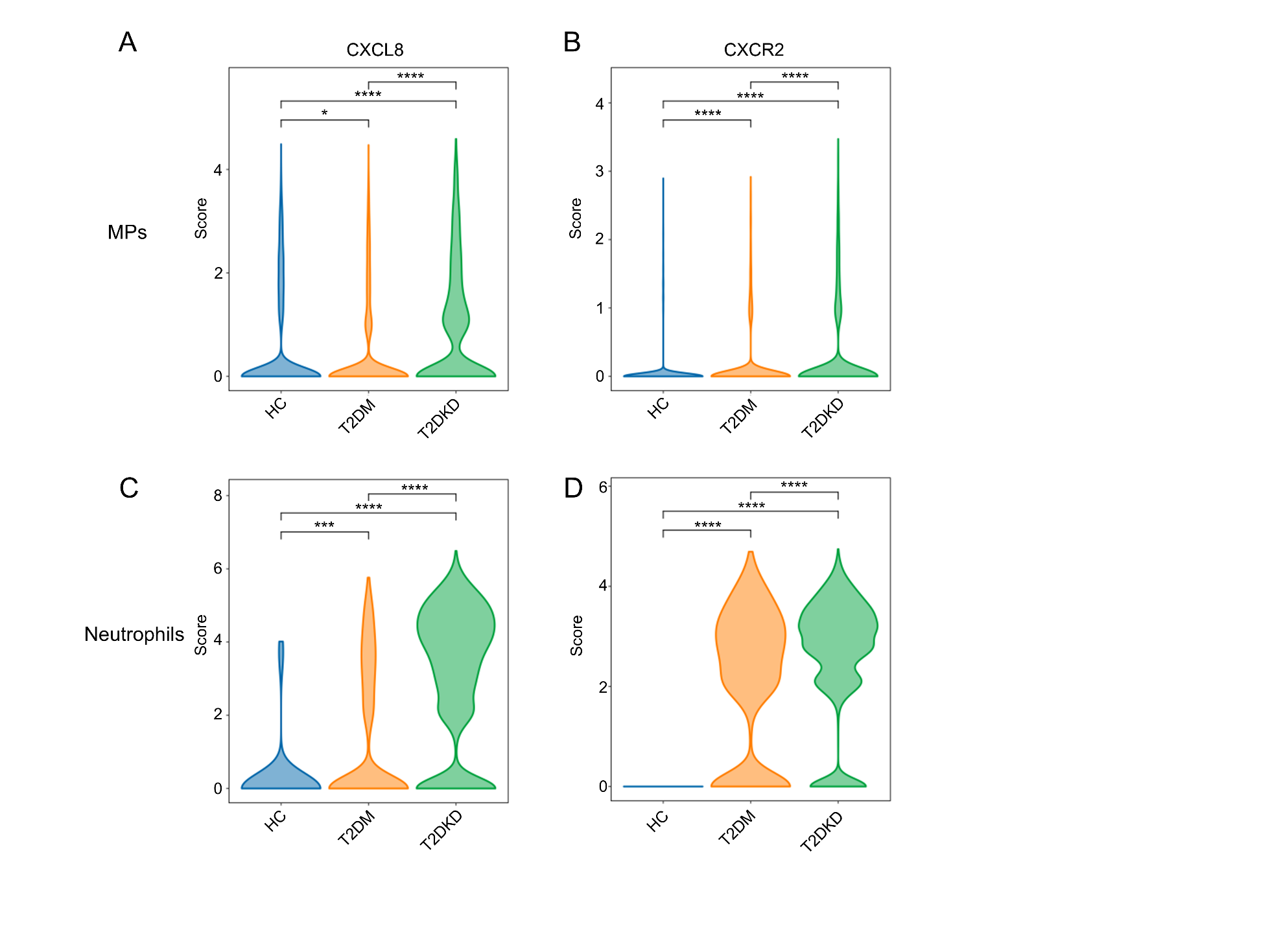


**Supplementary Figure 7. Expression of CXCL8 and CXCR2 in MPs and neutrophils across different groups**

A-B. Violin plots showed differences in the expression of CXCL8 (A) and CXCR2 (B) in MPs in different groups.

C-D. Violin plots showed differences in the expression of CXCL8 (C) and CXCR2 (D) in neutrophils in different groups.

**1.2 Supplementary Tables**

**Supplementary Table 1. Quality control metrics of scLacNAc-seq data**

| **Sample name** | **Cell number** | **Mean reads per cell** | **Median UMI per cell** | **Saturation (%)** | **Median gene per cell** | **Mt > 20%** | **Mt > 50%** |
| --- | --- | --- | --- | --- | --- | --- | --- |
| HC | 10112 | 29806 | 1842 | 81.65 | 888 | 1.99 | 0.11 |
| T2DM | 12313 | 25718 | 2197 | 75.86 | 1047 | 1.16 | 0.41 |
| T2DKD | 10783 | 29382 | 1708 | 78.48 | 751 | 0.69 | 0.29 |
| Mean | 11069 | 28302 | 1916 | 78.66 | 895 | 1.28 | 0.27 |

**Supplementary Table 2. Marker genes and percentage of primary cells identified by scLacNAc-seq analysis**

| **Cell clusters** | **markers** | **Cell numbers** | | | **Percentage (%)** | | |
| --- | --- | --- | --- | --- | --- | --- | --- |
|  |  | **HC** | **T2DM** | **T2DKD** | **HC** | **T2DM** | **T2DKD** |
| B cells | MS4A1 ^[1]^, CD79A ^[2]^, CD19 ^[3]^ | 674 | 641 | 106 | 7.97 | 5.86 | 1.08 |
| T and NK cells | CD3D ^[4]^, CD3E ^[5]^, CD3G ^[4]^ | 4228 | 5092 | 1764 | 49.97 | 46.54 | 17.91 |
| Neutrophils | FCGR3B [6], S100A9 ^[7]^, CSF3R ^[8]^ | 32 | 2198 | 5968 | 0.38 | 20.09 | 60.58 |
| Basophils | HDC ^[9]^, CLC ^[10]^, MS4A2 ^[11]^ | 9 | 89 | 14 | 0.11 | 0.81 | 0.14 |
| Mononuclear cells | CD14 ^[12]^, LYZ ^[13]^, HLA-DRA ^[14]^ | 3458 | 2792 | 1958 | 40.87 | 25.52 | 19.88 |
| Platelets | PPBP ^[15]^, PF4 ^[16]^, GP9 ^[17]^ | 60 | 130 | 41 | 0.71 | 1.19 | 0.42 |
| Total | N.A. | 8461 | 10942 | 9851 | 100 | 100 | 100 |

**Supplementary Table 3. Marker genes and percentage of sub-cell clusters identified by scLacNAc-seq analysis**

| **Cell clusters** | **markers** | **Cell numbers** | | | **Percentage (%)** | | |
| --- | --- | --- | --- | --- | --- | --- | --- |
|  |  | **HC** | **T2DM** | **T2DKD** | **HC** | **T2DM** | **T2DKD** |
| **Neutrophils** |  | | | | | | |
| CAMP | LTF, CAMP, LCN2 | 3 | 55 | 23 | 9.38 | 2.50 | 0.39 |
| CAMK1D | CAMK1D, EPHB1, RBM47 | 29 | 138 | 372 | 90.63 | 6.28 | 6.23 |
| FOLR3 | FOLR3, CST7, PROK2 | 0 | 231 | 1886 | 0.00 | 10.51 | 31.60 |
| PI3 | PI3, AIF1, SLPI | 0 | 413 | 2430 | 0.00 | 18.79 | 40.72 |
| MMP9 | MMP9, ARG1, HMGB2 | 0 | 979 | 240 | 0.00 | 44.54 | 4.02 |
| ISG15 | ISG15, RSAD2, IFIT1 | 0 | 382 | 1017 | 0.00 | 17.38 | 17.04 |
| **Mononuclear cells** |  | | | | | | |
| Classical mononuclear cells | CD14, VCAN, S100A8 | 3079 | 2172 | 1415 | 88.35 | 77.79 | 72.27 |
| Non-classical mononuclear cells | FCGR3A, CDKN1C, SMIM25 | 261 | 507 | 415 | 7.49 | 18.16 | 21.20 |
| Conventional dendritic cells | CD1C, FCER1A, CLEC10A | 116 | 91 | 118 | 3.33 | 3.26 | 6.03 |
| Plasmacytoid dendritic cells | CLEC4C, IL3RA, LILRA4 | 29 | 22 | 10 | 0.83 | 0.79 | 0.51 |

**Supplementary Table 4. Marker genes and percentage of sub-cell clusters identified by scLacNAc-seq analysis**

# Fold changes in T2DM/T2DKD-related gene set scores among subcell clusters

|  | Fold changes in T2DM-related gene set scores | Fold changes in T2DM-related gene set scores |
| --- | --- | --- |
| FOLR3/CAMK1D | 1.09 | 1.03 |
| FOLR3/CAMP | 1.10 | 1.02 |
| FOLR3/ISG15 | 1.04 | 1.01 |
| FOLR3/MMP9 | 1.03 | 1.00 |
| PI3/CAMK1D | 1.07 | 1.02 |
| PI3/CAMP | 1.09 | 1.02 |
| PI3/ISG15 | 1.03 | 1.01 |
| PI3/MMP9 | 1.02 | 1.01 |

# Supplementary references

1. Mudd, T.W., Jr.; Lu, C.; Klement, J.D.; Liu, K. MS4A1 expression and function in T cells in the colorectal cancer tumor microenvironment. *Cell Immunol* **2021**, *360*, 104260, doi:10.1016/j.cellimm.2020.104260.

2. Mason, D.Y.; Cordell, J.L.; Brown, M.H.; Borst, J.; Jones, M.; Pulford, K.; Jaffe, E.; Ralfkiaer, E.; Dallenbach, F.; Stein, H.; et al. CD79a: a novel marker for B-cell neoplasms in routinely processed tissue samples. *Blood* **1995**, *86*, 1453-1459.

3. Delage, L.; Manzoni, D.; Quinquenet, C.; Fontaine, J.; Maarek, A.; Chabane, K.; Mosnier, I.; Hayette, S.; Callet-Bauchu, E.; Grange, B.; et al. Molecular analysis of a CD19-negative diffuse large B-cell lymphoma. *Haematologica* **2019**, *104*, e114-e116, doi:10.3324/haematol.2018.203521.

4. Andreatta, M.; Corria-Osorio, J.; Müller, S.; Cubas, R.; Coukos, G.; Carmona, S.J. Interpretation of T cell states from single-cell transcriptomics data using reference atlases. *Nature Communications* **2021**, *12*, 2965, doi:10.1038/s41467-021-23324-4.

5. Liu, X.; Zhu, Z.; Wang, X. Specificity and function of T cell subset identities using single-cell sequencing. *Clinical and Translational Discovery* **2023**, *3*, e199, doi:<https://doi.org/10.1002/ctd2.199>.

6. Wang, Y.; Jönsson, F. Expression, Role, and Regulation of Neutrophil Fcγ Receptors. *Frontiers in Immunology* **2019**, *10*, doi:10.3389/fimmu.2019.01958.

7. Sprenkeler, E.G.G.; Zandstra, J.; van Kleef, N.D.; Goetschalckx, I.; Verstegen, B.; Aarts, C.E.M.; Janssen, H.; Tool, A.T.J.; van Mierlo, G.; van Bruggen, R.; et al. S100A8/A9 Is a Marker for the Release of Neutrophil Extracellular Traps and Induces Neutrophil Activation. *Cells* **2022**, *11*, doi:10.3390/cells11020236.

8. Dwivedi, P.; Greis, K.D. Granulocyte colony-stimulating factor receptor signaling in severe congenital neutropenia, chronic neutrophilic leukemia, and related malignancies. *Experimental Hematology* **2017**, *46*, 9-20, doi:<https://doi.org/10.1016/j.exphem.2016.10.008>.

9. Moriguchi, T.; Takai, J. Histamine and histidine decarboxylase: Immunomodulatory functions and regulatory mechanisms. *Genes to Cells* **2020**, *25*, 443-449, doi:<https://doi.org/10.1111/gtc.12774>.

10. Dvorak, A.M.; Letourneau, L.; Login, G.R.; Weller, P.F.; Ackerman, S.J. Ultrastructural Localization of the Charcot-Leyden Crystal Protein (Lysophospholipase) to a Distinct Crystalloid-Free Granule Population in Mature Human Eosinophils. *Blood* **1988**, *72*, 150-158, doi:<https://doi.org/10.1182/blood.V72.1.150.150>.

11. Klein, O.; Ngo-Nyekel, F.; Stefanache, T.; Torres, R.; Salomonsson, M.; Hallgren, J.; Rådinger, M.; Bambouskova, M.; Campbell, M.; Cohen-Mor, S.; et al. Identification of Biological and Pharmaceutical Mast Cell- and Basophil-Related Targets. *Scandinavian Journal of Immunology* **2016**, *83*, 465-472, doi:<https://doi.org/10.1111/sji.12436>.

12. Kuwana, M.; Okazaki, Y.; Kodama, H.; Izumi, K.; Yasuoka, H.; Ogawa, Y.; Kawakami, Y.; Ikeda, Y. Human circulating CD14+ monocytes as a source of progenitors that exhibit mesenchymal cell differentiation. *J Leukoc Biol* **2003**, *74*, 833-845, doi:10.1189/jlb.0403170.

13. Gu, Z.; Wang, L.; Dong, Q.; Xu, K.; Ye, J.; Shao, X.; Yang, S.; Lu, C.; Chang, C.; Hou, Y.; et al. Aberrant LYZ expression in tumor cells serves as the potential biomarker and target for HCC and promotes tumor progression via csGRP78. *Proc Natl Acad Sci U S A* **2023**, *120*, e2215744120, doi:10.1073/pnas.2215744120.

14. Mueller, A.; Kreuzfelder, E.; Nyadu, B.; Lindemann, M.; Rebmannn, V.; Majetschak, M.; Obertacke, U.; Schade, U.F.; Nast-Kolb, D.; Grosse-Wilde, H. Human leukocyte antigen-DR expression in peripheral blood mononuclear cells from healthy donors influenced by the sera of injured patients prone to severe sepsis. *Intensive Care Med* **2003**, *29*, 2285-2290, doi:10.1007/s00134-003-1992-8.

15. Smith, N.L.D.; Bromley, M.J.; Denning, D.W.; Simpson, A.; Bowyer, P. Elevated Levels of the Neutrophil Chemoattractant Pro–Platelet Basic Protein in Macrophages From Individuals With Chronic and Allergic Aspergillosis. *The Journal of Infectious Diseases* **2015**, *211*, 651-660, doi:10.1093/infdis/jiu490.

16. Buka, R.J.; Montague, S.J.; Moran, L.A.; Martin, E.M.; Slater, A.; Watson, S.P.; Nicolson, P.L.R. PF4 activates the c-Mpl–Jak2 pathway in platelets. *Blood* **2024**, *143*, 64-69, doi:10.1182/blood.2023020872.

17. Ghalloussi, D.; Rousset-Rouvière, C.; Popovici, C.; Garaix, F.; Saut, N.; Saultier, P.; Tsimaratos, M.; Chambost, H.; Alessi, M.-C.; Baccini, V. Bernard–Soulier syndrome: first human case due to a homozygous deletion of GP9 gene. *British Journal of Haematology* **2020**, *188*, e87-e90, doi:<https://doi.org/10.1111/bjh.16374>.
